# Supplementary material for: Quantum dynamical effects of vibrational strong coupling in chemical reactivity
Source: Nat Commun. 2023 May 12;14:2733. doi: 10.1038/s41467-023-38368-x (PMC10182063; doi:10.1038/s41467-023-38368-x)
Supplement: Supplementary file 1 — Supplementary Information [file 41467_2023_38368_MOESM1_ESM.pdf]

Supplementary Information: Quantum dynamical effects of vibrational strong coupling in chemical reactivity

Lachlan P. Lindoy[1], Arkajit Mandal[1], David R. Reichman[1] *

April 14, 2023

[1] Department of Chemistry, Columbia University, 3000 Broadway, New York, New York, 10027, U.S.A

Supplementary Note 1: Details of Molecular Systems

In the main-text we consider a one dimensional reaction coordinate coupled to a set of dissipative modes, such as Q and $\{X_j\}$. A set of secondary dissipative modes $\{x_j\}$ and $\{\mathcal{Q}_k\}$ are also coupled to the spectator mode Q and the cavity mode q_c. In the main text we consider three Debye baths that are coupled: to the reaction coordinate described by the spectral density $J_U(\Omega) = 2\Lambda_s\Omega\Gamma/(\Omega^2+\Gamma^2)$ (the solvent bath), to the spectator mode described by the spectral density $J_u(\omega) = 2\lambda_s\omega\gamma/(\omega^2+\gamma^2)$, and to the cavity mode described by the spectral density $J_L(\omega) = 2\lambda_L\omega\gamma_L/(\omega^2+\gamma_L^2)$ (the cavity loss bath). In the main-text the reorganization energy of the reaction coordinate bath, Λ_s, was obtained by setting the solvent friction, η_s, associated with the bath $\Lambda_s = \Gamma\eta_s/2$. The bath reorganization energies for the four friction values considered in Fig. 3c of the main text are provided in the Supplementary Table 1.

η_s	$0.02\omega_b$	$0.1\omega_b$	$0.5\omega_b$	$1.5\omega_b$
Λ_s	4.15×10^{-8} a.u.	2.08×10^{-7} a.u.	1.04×10^{-6} a.u.	3.11×10^{-6} a.u.

Supplementary Table 1: Numerical value (in atomic units) of bath reorganization energy Λ_s at various bath friction η_s, where ω_b is the reaction coordinate barrier frequency.

The reorganization energy for the cavity loss bath was obtained by setting the cavity loss parameter, τ_c, to $1/\tau_c = 2J_L(\omega_c)/(1-e^{-\beta\omega_c})$, and as such the reorganization energy for cavity loss depends on both the cavity loss parameter and the cavity frequency, ω_c. All other parameters, are in the the Supplementary Table 2.

λ_s	Γ	γ	γ_L
6.70×10^{-7} a.u.	200 cm^{-1}	1000 cm^{-1}	1000 cm^{-1}

Supplementary Table 2: Bath parameters used in this work. λ_s is the reorganization energy of the bath coupled to the spectactor mode and γ the cutoff frequency of this bath, Γ is the cutoff frequency of the solvent bath, and γ_L is the cutoff frequency of the cavity loss bath.

*drr2103@columbia.edu

Supplementary Note 2: Additional Absorption Spectra

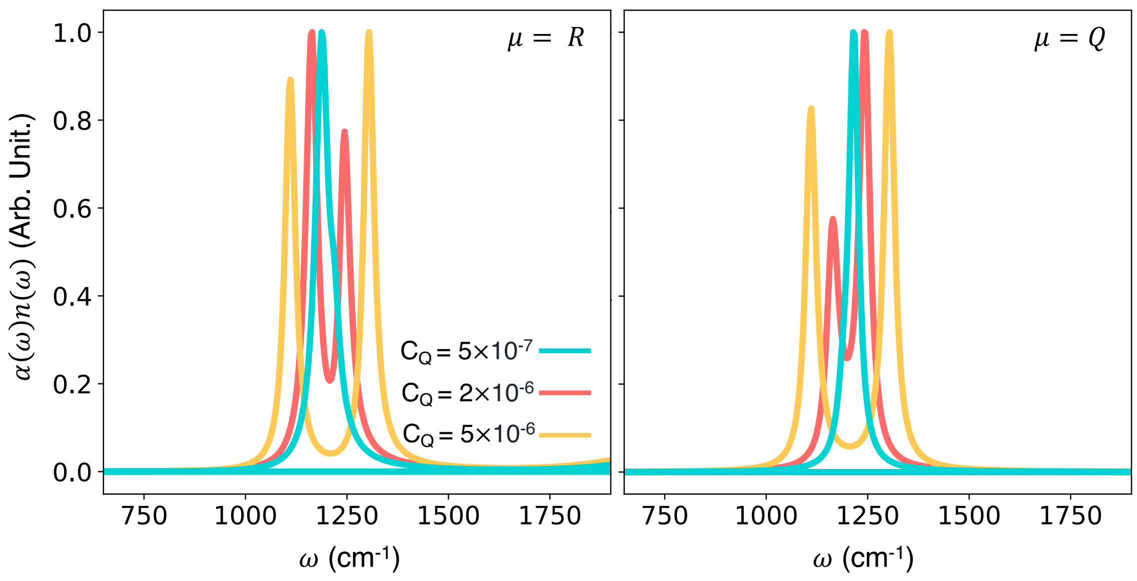

Supplementary Figure S1: Out-of-cavity absorption spectra $\alpha(\omega)n(\omega)$ as a function of frequency ω for the spectator mode models considered in Fig. 4 of the main text at three different spectator mode-reaction coordinate coupling C_Q. In each panel we have denoted the form of the dipole moment operator of the matter degrees of freedom μ that the cavity photon mode couples to. In the left panel the cavity photon mode is coupled to reaction coordinate R and in the right panel to the spectator mode coordinate Q. Source data are provided as a Source Data file.

In Fig. S1 we present the absorption spectra for the spectactor mode models considered in Fig. 4 of the main text. We have a total of six distinct spectra corresponding to the three different values for the spectator mode-reaction coordinate coupling, C_Q, and for the two different choices for the dipole operator $\hat{\mu} = \hat{R}$ or $\hat{Q}$. For $C_Q = 5 \times 10^{-7}$ a.u., a single peak with a small shoulder is observed, for all other cases the interactions between the near resonant reaction coordinate and spectator mode gives rise to a Rabi-splitting of the peaks that is observable in the absorption spectrum.

Supplementary Note 3: Various Molecular Systems

In this section we provide additional numerical results considering additional parameter regimes in addition to those considered in the main text.

Additional Unstructured Spectral Density Results

Low Frequency Symmetric Model Potential

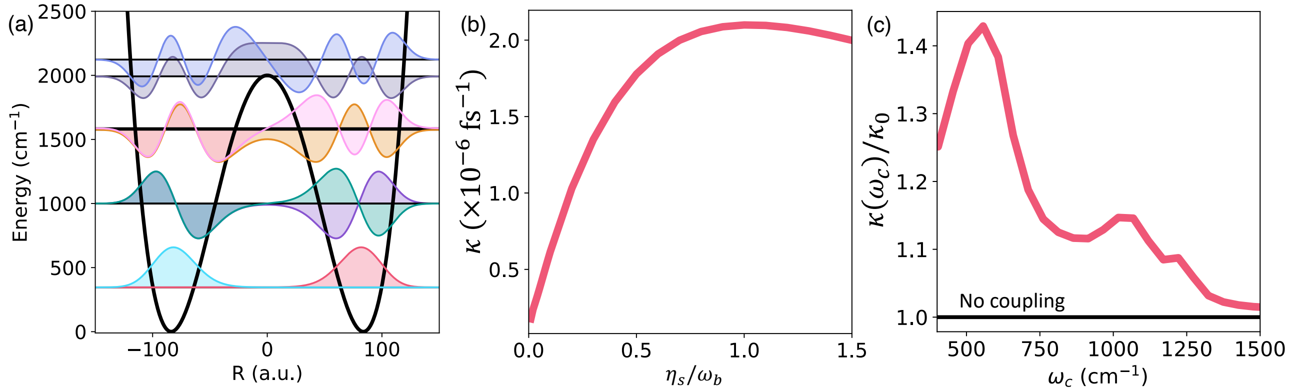

Supplementary Figure S2: **Cavity modification of ground state chemical kinetics.** (**a**) Potential energy surfaces as a function of reaction coordinate R for molecular ground adiabatic state with vibrational eigenstates. (**b**) Chemical rate constant κ as function of solvent friction η_s scaled by the barrier frequency ω_b. (**c**) Cavity modified chemical rate constant $\kappa(\omega_c)$ as function of cavity photon frequency ω_c. Here, κ_0 is the chemical rate constant outside cavity. Source data are provided as a Source Data file.

In Supplementary Fig. S2, we present cavity frequency-dependent rate profiles obtained for a model with an unstructured spectral density with friction constant $\eta_s = 0.1\omega_b$ and for a symmetric reaction coordinate potential with a lower barrier frequency $E_b = 2000$ cm^{-1} and lower well frequency, $\omega_b = 500$ cm^{-1} than considered in the main text. As shown in Supplementary Fig. S2a, this potential supports three vibrational modes in each well below the barrier. The rate constant as a function of the bath friction is shown in Supplementary Fig. S2b. Here we see the characteristic Kramer's turnover, and in contrast to the results in the main text we observe no tunneling dominated regime at low friction. The cavity frequency dependent rate constant, obtained with $\eta_c = 0.005$ a.u. and a cavity loss of $\tau_c = 1000$ cm^{-1}, shown in Supplementary Fig. S2c shows a number of distinct peaks

High Frequency Symmetric Model Potential

In Supplementary Fig. S3 we present cavity frequency-dependent rate profiles obtained for a model with an unstructured spectral density with friction constant $\eta_s = 0.1\omega_b$ and for a symmetric reaction coordinate potential with a higher barrier frequency $E_b = 2500$ cm^{-1} and higher well frequency, $\omega_b = 1500$ cm^{-1} than considered in the main text. As shown in Supplementary Fig. S3a, this potential supports only a single vibrational modes in each well below the barrier, with the first excited modes being found above the barrier. The rate constant as a function of the bath friction is shown in Supplementary Fig. S3b, here we see a sharp decrease in the rate constant with increasing rate constant that is characteristic of the tunneling dominated regime [1]. Additionally, even at large friction constants, past the Kramer's turnover point for the other models, we do not see any turnover, instead we observe a decrease in the rate constant with increasing friction. For this model, no significant, resonant cavity modification of the reaction rate is observed. As shown in Supplementary Fig. S3c, where we have included a cavity mode that couples to the system with $\eta_c = 0.005$ a.u. and a cavity loss of $\tau_c = 1000$ cm^{-1}, the presence of the cavity mode leads to an off-resonant suppression as has been discussed in the main text.

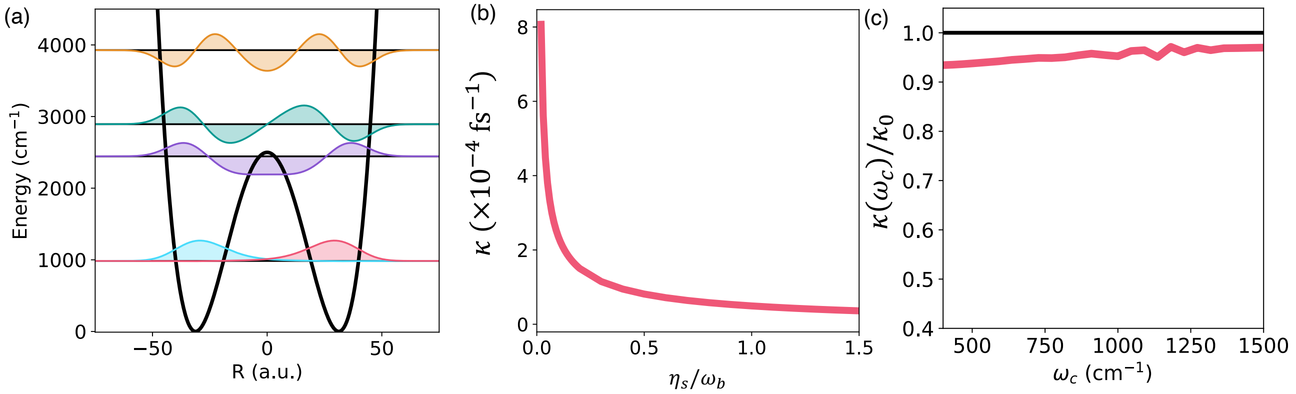

Supplementary Figure S3: **Cavity modification of ground state chemical kinetics.** (**a**) Potential energy surfaces as a function of reaction coordinate R for molecular ground adiabatic state with vibrational eigenstates. (**b**) Chemical rate constant κ as function of solvent friction η_s scaled by the barrier frequency ω_b. (**c**) Cavity modified chemical rate constant $\kappa(\omega_c)$ as function of cavity photon frequency ω_c. Here, κ_0 is the chemical rate constant outside cavity. Source data are provided as a Source Data file.

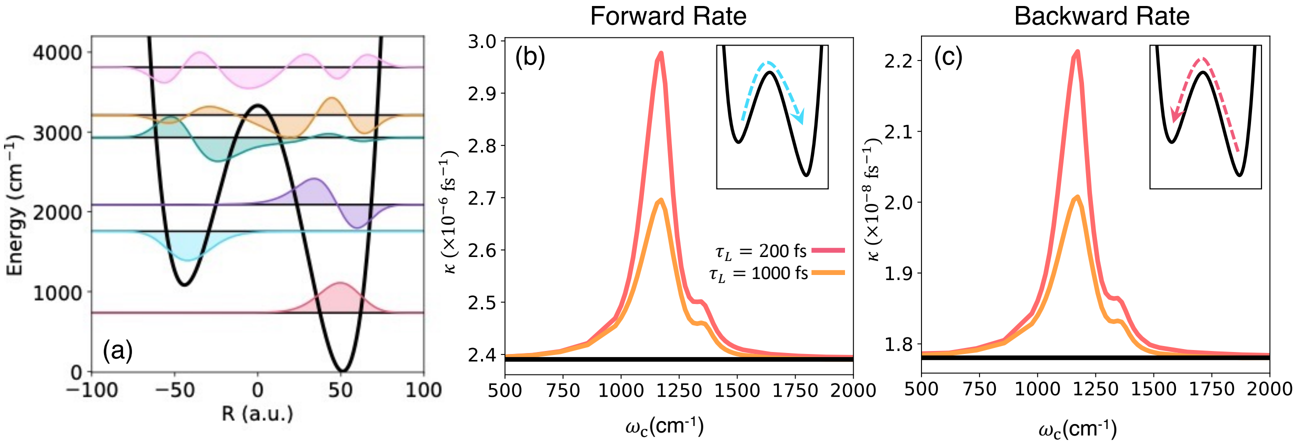

Supplementary Figure S4: **Cavity modification of ground state chemical kinetics in an asymmetric model system.** (**a**) Potential energy surfaces as a function of reaction coordinate R for molecular ground adiabatic state with vibrational eigenstates. Cavity modified (**b**) forward and (**c**) backward chemical rate constant κ as function of cavity photon frequency ω_c. The black solid line represents the (**b**) forward and (**c**) backward chemical rate constant outside cavity. Source data are provided as a Source Data file.

Asymmetric Model Potential

In Supplementary Fig. S4 we present cavity frequency-dependent rate profiles obtained for a model with an unstructured spectral density with friction constant $\eta_s = 0.1\omega_b$ and for an asymmetric reaction coordinate potential with $\omega_b = 1030$ cm^{-1}, $E_b = 2730$ cm^{-1}, and $c = 2.27817 \times 10^{-8}$ a.u. This choice of parameters lead to vibrational transition frequencies in the reactant well that do not differ significantly from those considered in the main text. We have considered a lossy cavity mode with $\tau_c = 1000$ cm^{-1} and $\eta_c = 0.00125$ a.u. In contrast to the results in the main text, here we observe a small shoulder beyond the main peak in the rate profile, this shoulder occurs at a frequency corresponding to the transition between the ground and first excited state of the product well, and can be attributed to a cavity enhancement of the energy loss associated with product well. It is worth noting that this peak is considerably smaller than that observed for the reactant well, and this can additionally be rationalized in terms of energy loss-based arguments. In the absence of the cavity, the energy loss associated with the product

well is considerably larger than that associated with the reactant well (owing to the increased well depth). As the rate profile becomes exponentially insensitive to cavity loss in the high cavity loss limit [2], the larger starting loss associated with the product well leads to a less significant cavity enhancement.

Additional Structured Spectral Density Results

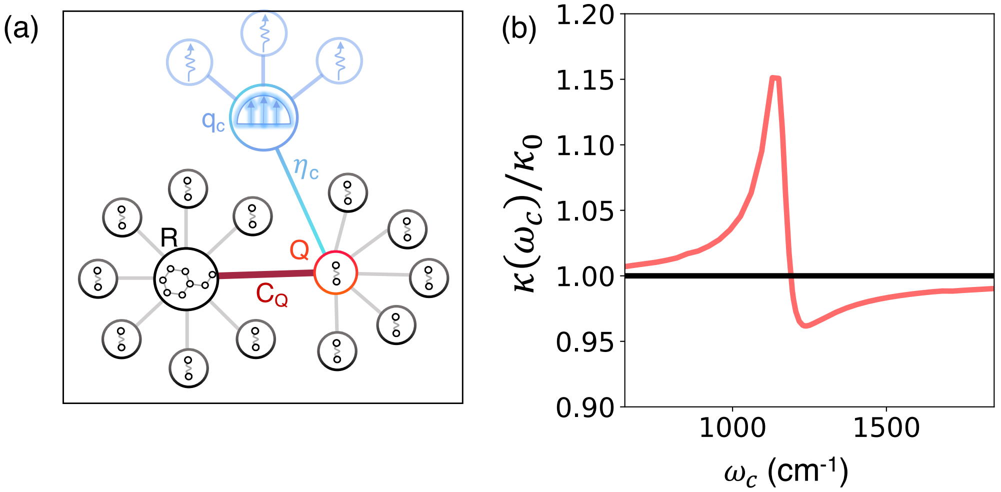

Supplementary Figure S5: **Cavity modification of ground state chemical kinetics.** (**a**) Schematic illustration of a cavity-molecule-solvent setup, with the cavity q_c coupling to the spectator mode Q with strength η_c. This spectator mode couples to the reaction coordinate R with strength C_Q. (**b**) Cavity modified chemical rate constant $\kappa(\omega_c)$ as function of cavity photon frequency ω_c. Here, κ_0 is the chemical rate constant outside cavity. Source data are provided as a Source Data file.

Finally we consider a system with a structured spectral density arising from the presence of an underdamped spectator mode that couples to the reaction coordinate. This model was considered in Supplementary Fig. 4 of the main text, however, here we will consider a lower frequency spectator mode with $\omega_Q = 1100$ cm^{-1}. In Supplementary Fig. S5, we present the cavity frequency dependent rate profile obtained for this model when a lossy cavity mode, with $\tau_c = 1000$ cm^{-1} is coupled, with $\eta_c = 0.005$ a.u., to the spectator mode (the case considered in Supplementary Fig. 4 (d-f) of the main text).

Supplementary Note 4: Classical Spectra and Dynamics

In this work, the classical absorption is obtained by computing the position-position correlation [3]

$$\alpha(\omega)n(\omega) \propto \lim_{t_f \to \infty} \omega(1 - \exp(-\beta\omega)) \int_{-t_f}^{t_f} \langle R(t)R(0)\rangle e^{-i\omega t}, \tag{1}$$

where t_f is the duration of the simulation. Note that $\langle R(t)R(0)\rangle = -\langle R(-t)R(0)\rangle$ is antisymmetric.

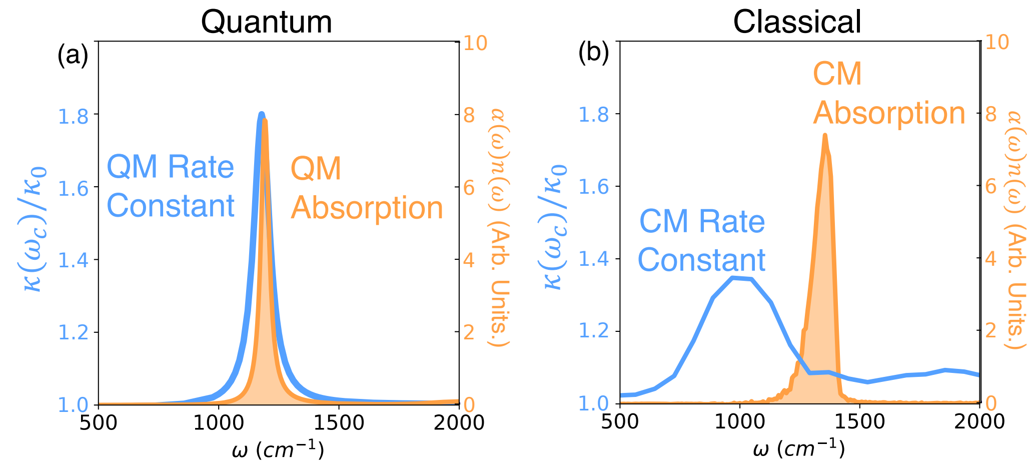

Supplementary Figure S6: **Quantum versus Classical dynamics.** Cavity frequency dependent chemical rate constant $\kappa(\omega_c)$ (κ_0 is the rate constant outside cavity) as a function of photon frequency $\omega = \omega_c$ as well as absorption $\alpha(\omega)n(\omega)$ as a function frequency ω obtained from (**a**) exact quantum dynamics and (**b**) classical dynamics at various cavity lifetimes for the same model as Fig. 2 in the main-text. (**c**) Chemical rate constant at a photon frequency that leads to maximum modification at various cavity lifetimes. Classical results are obtained with $\omega_c = 967.9$ cm^{-1} and quantum results are obtained with $\omega_c = 1185.7$ cm^{-1}.

In Figs. 2 and 3 of the main text, we have considered how various model parameters influence the cavity modification of the chemical reaction rate, obtained quantum mechanically, for the unstructured solvent model. Here, we will repeat this, but now consider the classical rate profiles. We start by comparing the rate profiles obtained using classical and quantum mechanical calculations to the corresponding molecular (out of cavity) absorption spectra. As shown in Supplementary Fig. S6a (Fig. 2d of the main text), the quantum rate profile exhibits a sharp peak that occurs very close to resonance ($\lesssim 10$ cm^{-1}), from the peak. Additionally, while the peak in the rate profile is broader than the absorption peak, it is of a comparable width. In comparison, the classical treatment leads to vastly different results. In particular, the peak in the rate profile is observed at a cavity frequency of ~ 1000 cm^{-1}, while the absorption spectrum is peaked at ~ 1400 cm^{-1}, near the harmonic well frequency of 1414 cm^{-1}. In contrast to the absorption profile, which is dominated by "bottom of the well properties", the classical rate profile is dominated by energy loss processes that occur for trajectories transitioning between barrier and well regimes [2]. As such, while this does depend on the well frequency [4], it is significantly influenced by anharmonicity in the potential near the barrier, and is thus not observed at resonant with the absorption spectrum peak.
We next consider the effect of cavity loss on the classical rate profile. As shown in Supplementary Fig. S7b, the rate profile obtained from classical mechanics shows little sensitivity to cavity loss compared to the quantum results from the main text (reproduced in Supplementary Fig. S7a for ease of comparison) for the values of the cavity loss rate considered here. This difference in the sensitivity to cavity loss between the two approaches is further illustrated in Supplementary Fig. S7c), where the peak enhancement of the reaction rate is shown as a function of the cavity loss. In contrast to the quantum mechanical results where cavity loss plays a vital role in determining the rate, the classical treatment predicts an enhancement at ~ 1000 cm^{-1}, in the presence of a perfect cavity, and shows far less sensitivity to loss.

Finally, we consider the effect of the light-matter coupling strength on the classical rate profile. As shown in

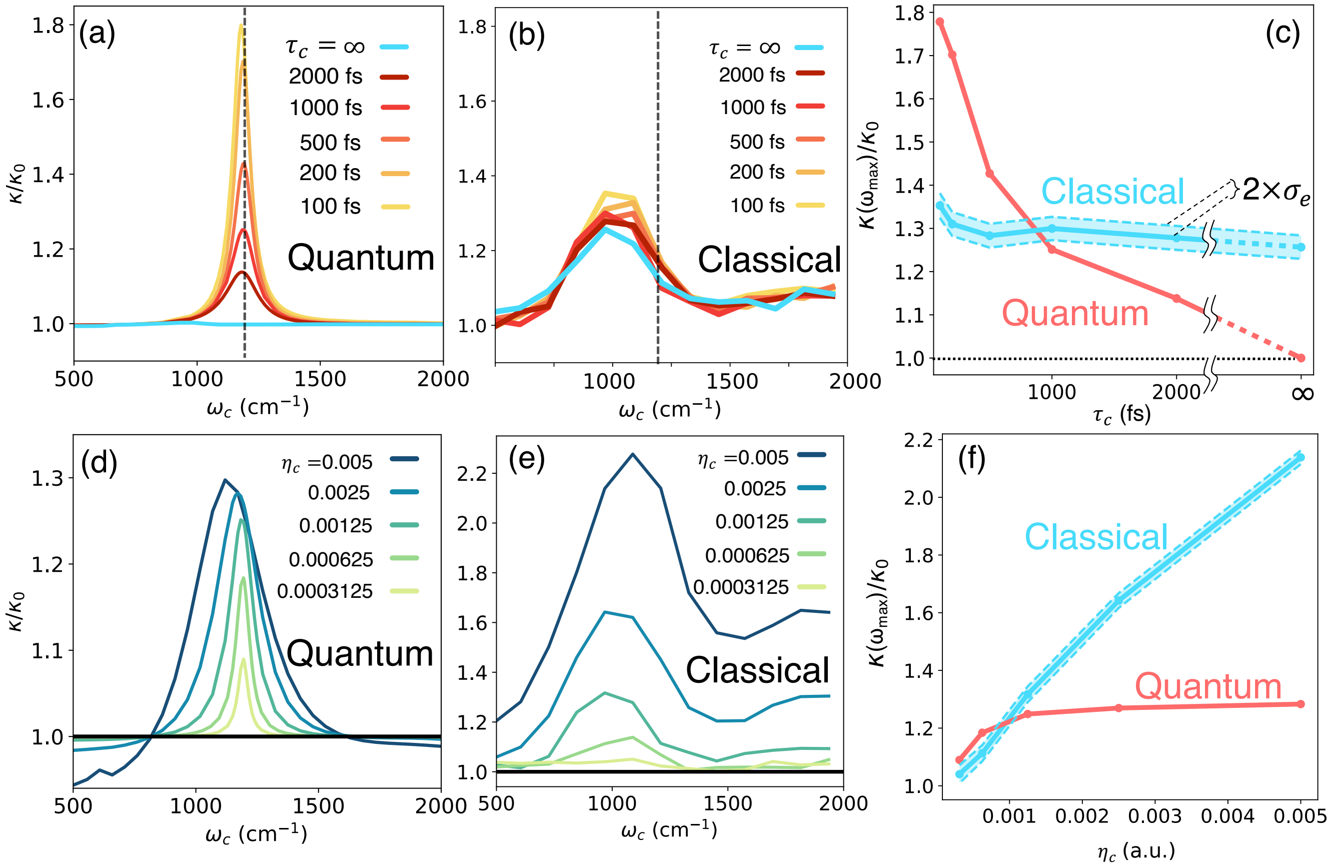

Supplementary Figure S7: **Quantum versus Classical dynamics.** Cavity frequency dependent chemical rate constant $\kappa(\omega_c)$ (κ_0 is the rate constant outside cavity) as a function of photon frequency ω_c obtained from (**a**) exact quantum dynamics and (**b**) classical dynamics at various cavity lifetimes τ_c for the same model as Fig.2 in the main-text. The vertical black dashed line in (**a**) and (**b**) indicates the vibrational excitation energy of the bare molecular system. (**c**) Chemical rate constant at a photon frequency that leads to maximum modification at various cavity lifetimes. Classical results are obtained with ω_c = 967.9 cm−1 and quantum results are obtained with ω_c = 1185.7 cm−1. Cavity modified chemical rate constant asa function of the light-matter coupling obtained (d) quantum mechanically and (e) classically. (f) Chemical rate constant at a photon frequency that leads to maximum modification, similar to (c) but as a function of light-matter coupling. The shaded regions in panels (c) and (f) depict two times the standard error of the mean σ_e obtained from the 1,500,000 trajectories used to obtain the classical rate constants. Source data are provided as a Source Data file.

Supplementary Fig. S7e, the cavity induced enhancement of the chemical reactivity increases significantly with increasing coupling strength, while also becoming significantly broader, with significant (20%) enhancement observed far from resonance ($\omega_c = 500$ cm^{-1}) at the strongest light-matter coupling strength considered. Further, we observe the appearance of a significant second peak at $\omega_c \sim 2000$ cm^{-1}, which can likely be attributed to features arising from vibrational overtones, but is not observed in the quantum mechanical treatment (Supplementary Fig. S7d). Further, we note that the peak enhancement observed classically shows a significantly larger frequency dependence that the quantum mechanical results, increasing to a $\sim 220\%$ peak enhancement for $\eta_c = 0.005$ a.u. This is in stark contrast to the quantum mechanical results where the effect begins to saturate with strong system bath coupling and the peak enhancement becomes near independent of the light matter coupling strength between $\eta_c = 0.0025$ and 0.005 a.u. We note that similar saturation of the cavity modification of chemical reactivity has been observed in prior experiments [5] (with the caveat that these experiments show suppression rather than enhancement).
A total of 1,500,000 trajectories were ran to converge the classical rate calculations in this work. We present the rate profiles, and error bars showing two times the standard error in the mean associated with the 1,500,000 trajectories in Supplementary Fig. S8, for different values of the cavity loss.

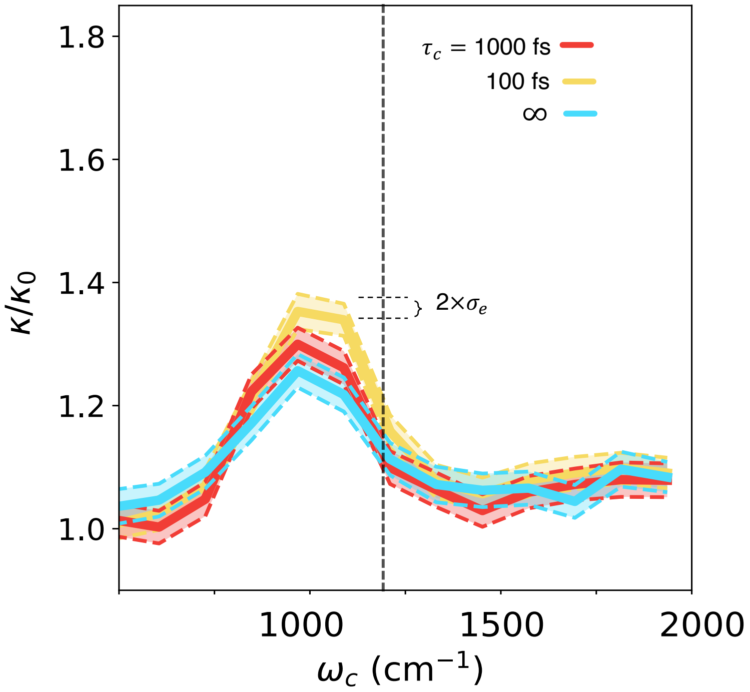

Supplementary Figure S8: **Standard error for computing classical transmission coefficient.** Cavity modified chemical rate constant κ as a function of the photon frequency ω_c with shaded area representing twice of the standard error σ_e. Here, κ_0 is the chemical rate constant outside cavity. The vertical black dashed line indicates the vibrational excitation energy of the bare molecular system. The standard error is estimated as $\sigma_e = \frac{1}{\sqrt{N}}\sqrt{\frac{\sum_i(\kappa_i - \langle\kappa\rangle)^2}{N}}$. Note that here we have used k_i as the transmission coefficient obtained by averaging over 5000 trajectories and we consider 300 such independent realization of k_i, such that we have a total of $1,500,000$ trajectories. Source data are provided as a Source Data file.

Supplementary Note 5: Effects of Cavity Loss on the Rate Constant

In Fig. S9, we consider properties of the time-dependent transmission coefficient obtained for the symmetric double well potential model considered in the main text with an unstructured spectral density with $\eta_s = 0.1\omega_b$, a light-matter coupling of $\eta_c = 0.00125$ (used to obtain Fig. 3b of the main text). In Supplementary Fig. S9a we present the time-dependent transmission coefficients obtained for this model in the absence of cavity loss. For a cavity frequency of $\omega_c = 1195$ cm^{-1} (near resonant with the reaction coordinate vibration), the transmission coefficient exhibits a significant short-time enhancement. During this period of time, significant oscillations observed in the instantaneous rate can be attributed to oscillations of the cavity mode. These oscillations decay with time as the system approaches thermal equilibrium. This decay of oscillations is correlated with a decay of the transmission coefficient, which approaches the results obtained off resonance in the long time limit.

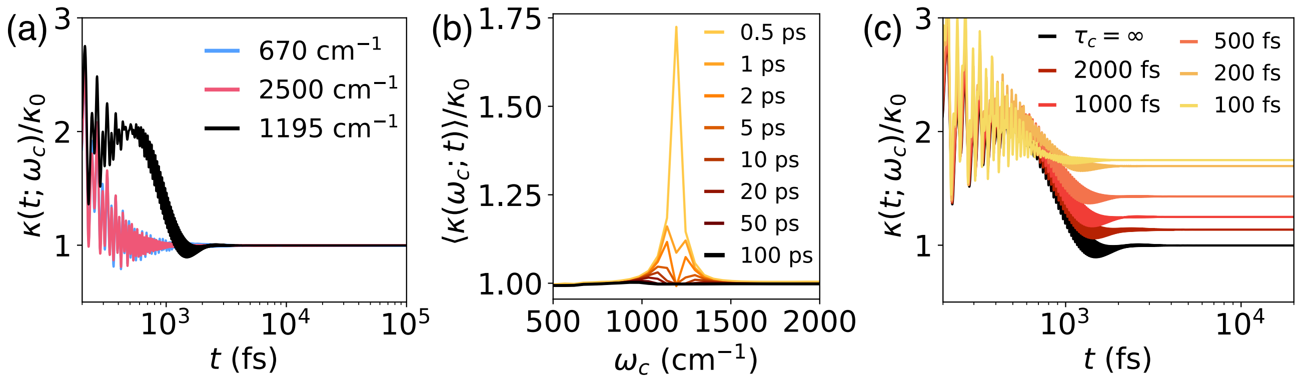

Supplementary Figure S9: **Effect of Cavity Loss on Instantaneous Transmission Coefficients and Reaction Rates.** (a) Time-dependent normalized instantaneous rate constants $\kappa(t, \omega_c)$ (κ_0 is the rate constant outside cavity) obtained in the absence of cavity loss for three different values of the cavity frequency. (b) The average, normalized, instantaneous rate constants obtained over a period of 500 fs starting at different points in time. (c) The time-dependent instantaneous rate constant obtained for a cavity frequency of 1195 cm^{-1} and with varying values of the cavity lifetime τ_c. All results here are obtained for the unstructured spectral density model with $\eta_s = 0.1\omega_b$ considered in the main text and with a light-matter coupling of $\eta_c = 0.00125$. In all cases the instantaneous rate constants are normalized by the out-of-cavity rate. Source data are provided as a Source Data file.

The initial enhancement of the instantaneous rate constant, and the decay of this enhancement as the cavity mode thermalizes, is further illustrated in Supplementary Fig. S9b. Here the average instantanous rate constant obtained over a period of 500 fs starting at different times is shown as a function of the cavity frequency. At short times, significant enhancement in the instantaneous rate constant are observed. At longer times this enhancement decays most rapidly at resonance with the feature splitting into two peaks, with maxima that occur at larger detuning from resonance at longer times.

Finally, in Supplementary Fig. S9c, the effect of cavity loss on the time-dependent instantaneous rate constant is shown. As the cavity loss is increased, the oscillations in the instantaneous rate constant decay more rapidly, indicating more rapid thermalization of the cavity mode. In addition, this increase in the rate of thermalization of the cavity mode coincides with an increase in the plateau value of the instantaneous rate constant, and therefore an enhancement of the thermal rate constant.

These effect can be understood by considering energy transfer processes between the reaction coordinate - cavity subsystem (containing only R and q_c and described by $\hat{H}_{\mathrm{mol}} + \hat{H}_{\mathrm{cav}}$) and its interaction with a dissipative bath composed of the solvent degrees of freedoms $\{X_j\}$ and the far-field cavity modes $\{\mathcal{Q}_k\}$. For the molecule-cavity system initially at equilibrium in the left well, the average cavity position will be displaced from equilibrium. Conversely, if the system was at equilibrium in the right well, the average cavity position will be displaced from equilibrium in the opposite direction. As such, reaction from the left to right will result in non-equilibrium, and generally high energy, configurations for the cavity mode. This process leads to a loss in energy from the reaction coordinate, which, in the low friction regime, results in an enhancement of the instantaneous rate constant.

In the absence of cavity loss, thermalization of the cavity mode will occur through energy transfer from the cavity mode to the molecular bath that is mediated by transfers between the cavity mode and reaction coordinate. This process depends sensitively on the detuning of the cavity mode and reaction coordinate frequency. For high energy cavity configurations, this process requires a transfer of energy from the cavity to the reaction coordinate decreasing the energy loss of the reaction coordinate, and in turn decreasing the rate constant.

Cavity loss provides an alternative pathway for thermalization of the cavity mode, enhancing the rate of thermalization. Additionally, this pathway does not require transfer of energy from the cavity mode to the reaction coordinate, and enables the cavity mode to act as an additional source of dissipation increasing the reaction rate in this regime.

Additionally, these results demonstrate that care should be taken when evaluating cavity-modified chemical reaction rates in the absence of cavity loss. Depending on the extent of detuning from resonance, the timescales associated with slow energy transfer processes can determine the timescale over which the rate constant plateaus.

Supplementary Note 6: Non-linear Dipole Operator

In the main text we have considered the case of bi-linear coupling between the molecular system and cavity mode. In doing so we have made an assumption of a linear dipole moment for the system, here we consider lifting this assumption and consider a non-linear dipole operator for the molecular system of the form

$$\hat{\mu} = \hat{R}e^{-d^2\hat{R}^2} \tag{2}$$

shown in Supplementary Fig. S10a).

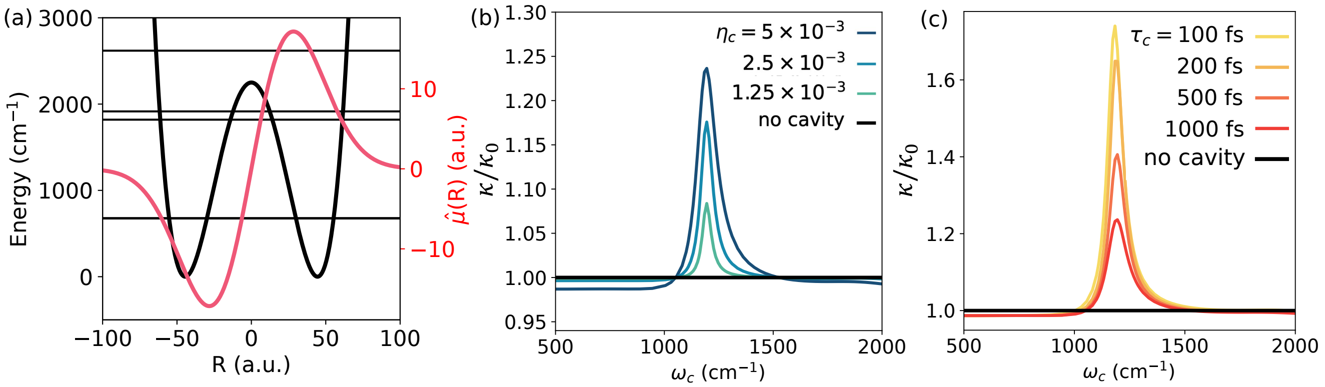

Supplementary Figure S10: **Cavity modification to chemical rate with nonlinear permanent dipole** (a) The potential energy surface as a function of the reaction coordinate R and eigenstates (black) and nonlinear permanent dipole moment $\mu(R)$ (red). (b) Effect of light-matter coupling η_c on chemical rate constant $\kappa(\omega_c)$ as a function of the cavity photon frequency ω_c with cavity lifetime $\tau_c = 1000$ fs and solvent friction $\eta_s = 0.1\omega_b$ and with a nonlinear dipole moment operator. (c) Effect of cavity lifetime τ_c at $\eta_c = 0.005$ and $\eta_s = 0.1\omega_b$. Source data are provided as a Source Data file.

Supplementary Fig. S10b presents the cavity modified rate constants at various light-matter coupling strengths η_c as a function of the cavity frequency for the unstructured spectral density model with cavity lifetime $\tau_c = 1000$ fs and solvent friction $\eta_s = 0.1\omega_b$. Here we observe similar results to those obtained for the linear dipole moment operator case (Fig. 3a of the main text), in which we see a sharp peak at resonance with the molecular vibrational transition. Here, however, we observe significant asymmetry in the peak structure arising from an increase in the dipole moment matrix element between the ground vibrational state and the higher of the two vibrationally excited states near the barrier.

In Supplementary Fig. S10c we revisit the question of the importance of cavity loss for the case of the nonlinear dipole. Once again, we observe that an increase in cavity loss gives rise to an increase in the height of the rate profile, demonstrating that this feature is robust to non-linearities in the light-matter interaction.

Supplementary Note 7: Models with Higher Energy Barriers

In the main-text we considered the dynamics of a model for which the reaction barrier was taken as 2250 cm^{-1}, corresponding to $\beta E_b \approx 10.8$. Here, in order to reduce the importance of "below the barrier tunnelling", we consider the effect of increasing the barrier height, while keeping the reaction coordinate frequency constant. In doing so we increase both the width and height of the reaction barrier, increasing the number of vibrational states below the barrier.

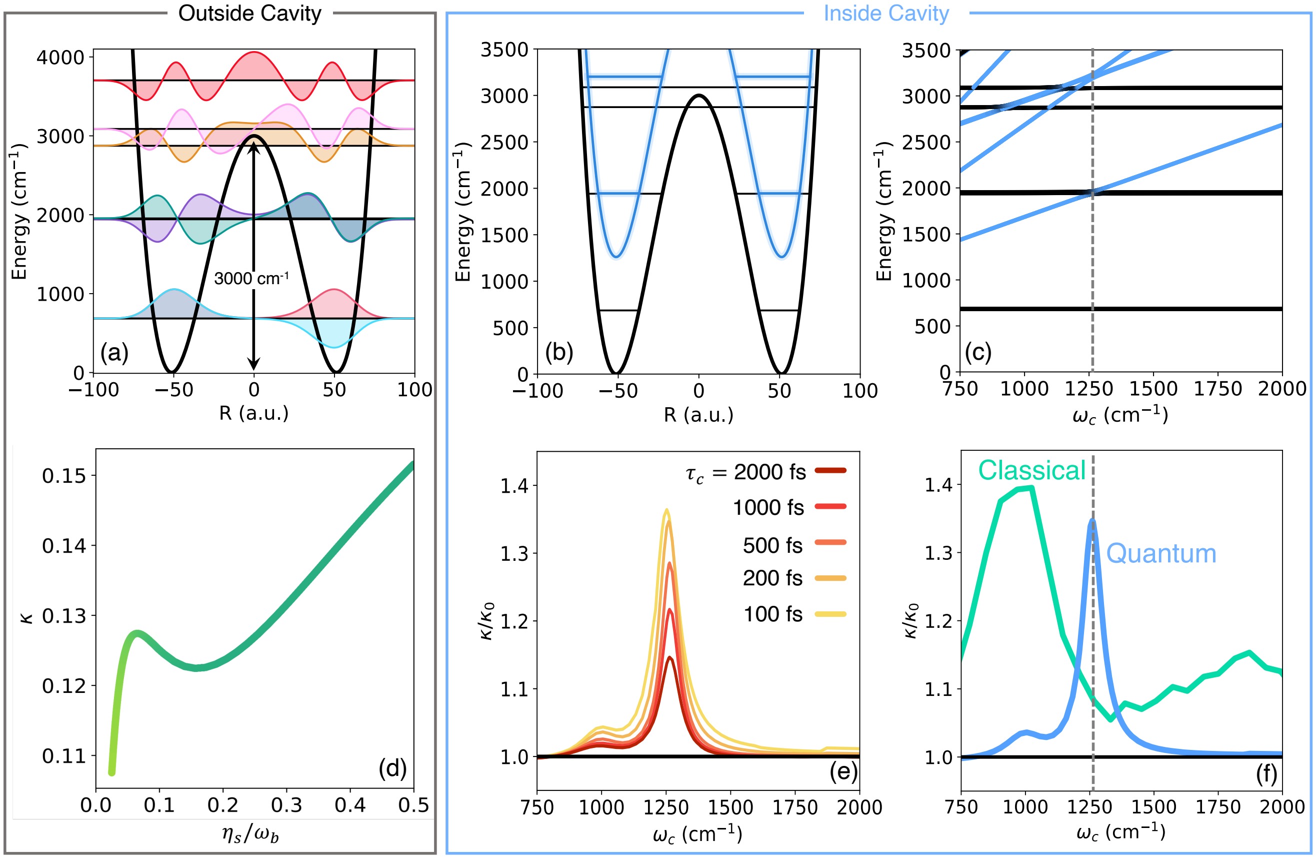

Supplementary Figure S11: **Higher energy barrier (3000 cm^{-1}) system.** (a) Potential energy surface as a function of the reaction coordinate R and vibrational eigenstates. (b) Potential energy surfaces for a molecular ground adiabatic state with 0 photons $|G, 0\rangle$ (black solid line) and with 1 photon (blue solid line) $|G, 1\rangle$, as well as the corresponding vibrational eigenstates of $|G, 0\rangle$ and $|G, 1\rangle$ (horizontal sold lines). (c) Vibrational polariton eigenspectrum as a function of cavity photon frequency ω_c. (d) Chemical rate constant κ as a function of solvent friction η when the molecule is embedded in an unstructured environment. (e) Effect of cavity lifetime τ_c on cavity modified chemical reactivity. (f) Comparing the cavity modified chemical rate constant κ (κ_0 is the chemical rate constant outside cavity) computed using exact quantum (blue solid line) and classical (green solid line) dynamical simulations. Source data are provided as a Source Data file.

We start by considering a model with $\omega_b = 1000$ cm^{-1} and a barrier of $\beta E_b = 3000$ cm^{-1}. As shown, in Fig. S11a, this model gives rise to two vibrational energy levels in each well with energies well below the barrier, with the 5th and 6th levels appearing near the barrier top. Compared to the model considered in the main text, we do not see a dramatic sudden increase in the rate constant at low friction, as shown in Fig. S11d, indicating a suppression of the tunnelling mechanism due to the larger reaction barrier.

In the presence of coupling to a cavity, we observe an avoided crossing in the polaritonic energy levels when the cavity frequency is near resonant with the first vibrational transition at 1250 cm^{-1}, as shown in Supplementary Fig. S11c. Consistent with the results presented in the main text, we observe an enhancement in the chemical reactivity that is peaked when the cavity frequency is resonant with the first vibrational transition, as shown in

Figs. S11e and S11f). Additionally, decreasing the cavity lifetime leads to more significant cavity modification of chemical reactivity, as shown in Supplementary Fig. S11e. Finally, as shown in Supplementary Fig. S11f, classical GLE simulations for this model are not able to accurately reproduce the quantum mechanical rate profiles. While the classical treatment does predict an enhancement in the rate profile, the observed profile is significantly broader (FWHM $\sim$ 400 cm^{-1}) compared to the quantum calculations (FWHM $\sim$ 85 cm^{-1}). Additionally, The use of a classical treatment gives rise to a significant detuning ($>$ 250 cm^{-1}) in the location of the peak enhancement of the rate. Here, all results in the presence of a cavity were obtained with a solvent friction constant $\eta_s = 0.075\omega_b$.

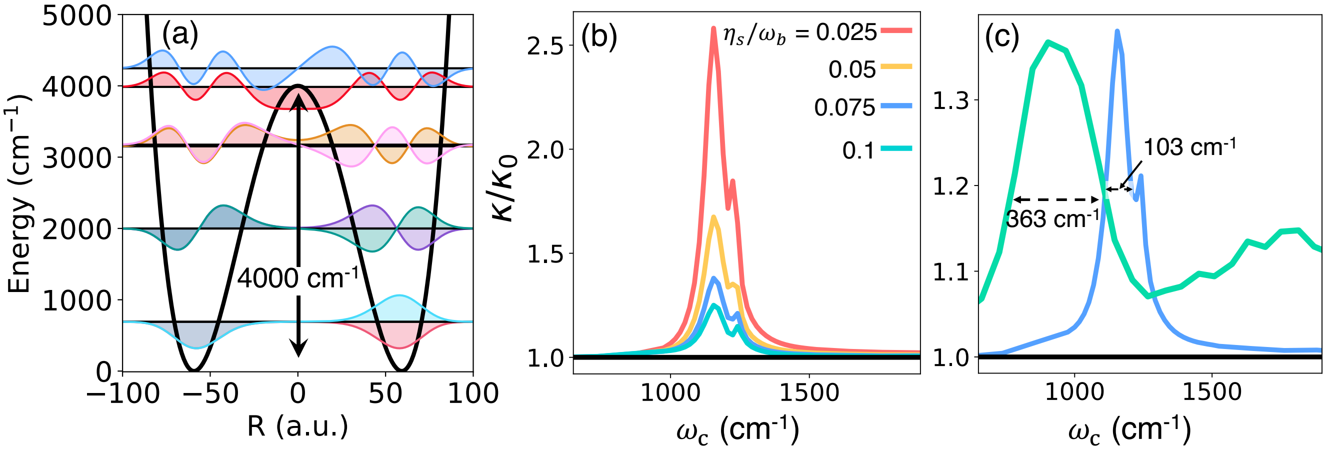

Supplementary Figure S12: **Higher energy barrier (4000 cm^{-1}) system.** (a) Potential energy surface as a function of the reaction coordinate R and vibrational eigenstates. (b) Cavity modification of chemical rate constant κ (κ_0 is the chemical rate constant outside cavity) at various solvent friction η_s (scaled by the barrier frequency ω_b). (c) Comparing the cavity modified chemical rate constant computed using exact quantum (blue solid line) and classical (green solid line) dynamical simulations. Source data are provided as a Source Data file.

In Fig. S12, we consider a model with further increased barrier height $E_b = 4000$ cm^{-1}, with $\omega_b = 1000$ cm^{-1} and $\tau_c = 200$ fs. As shown in Supplementary Fig. S12a, in this regime we have three vibrational levels well below the barrier in each well, with the 7th and 8th levels appearing near the barrier top. In the presence of coupling to a cavity, we observe an enhancement in the chemical reactivity that exhibits peaks at cavity frequencies corresponding to the transition between the ground and first vibrational states and between the first and second vibrational states in the reactant well, as shown in Fig S12b. In this figure, we compare the cavity enhancement observed for varying strengths of the solvent friction, and observe that the extent of the enhancement observed is strongly dependent on the solvent friction.

Finally, in Supplementary Fig. S12c, we compare the results obtained from classical and quantum mechanical simulations for the case of $\eta_s = 0.075\omega_b$. Here we observe that as for the other models considered in the text, the classical results exhibit a significantly broader peak (FWHM of $\sim$ 360 cm^{-1}) compared to the quantum mechanical results (in which we observe two separate peaks, with the larger of the two having a FWHM of $\sim$ 100 cm^{-1}). Further the classical results exhibit significant red-shifting of the peak position, entirely consistent with the results presented in the main text.

Supplementary Note 8: Effect of Off-Resonant Peak in structured Spectral Density

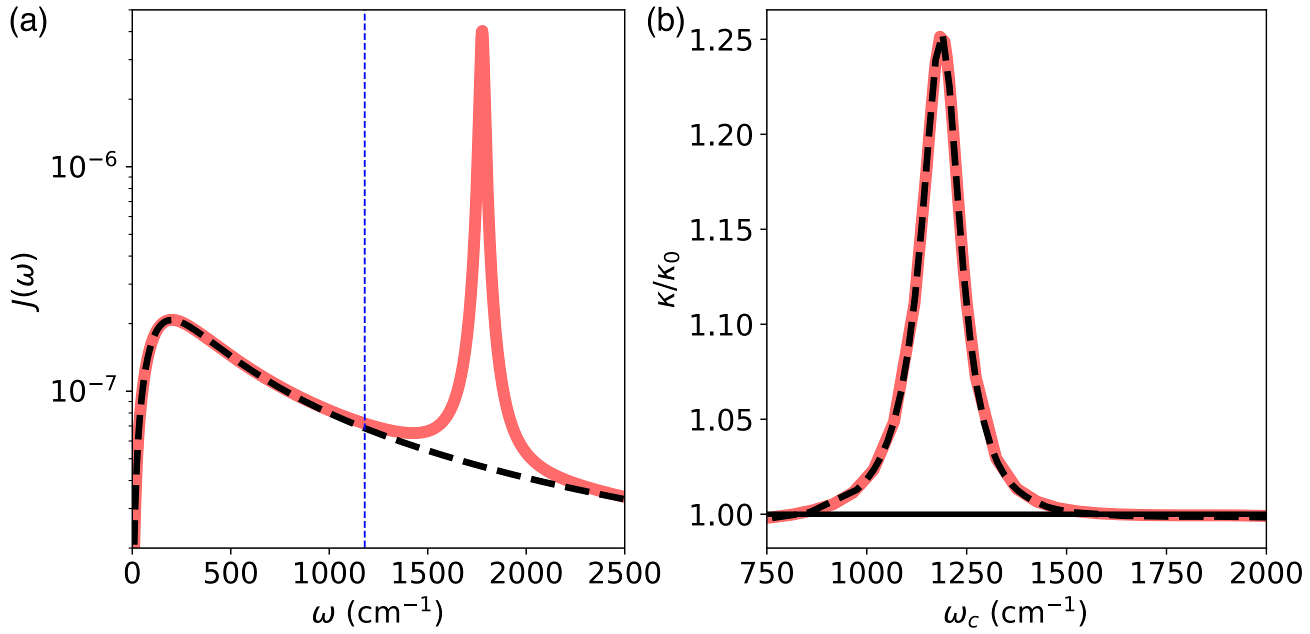

Supplementary Figure S13: **Structured solvent spectral density with off-resonant peak.** (a) Unstructured (black) and Structured (red) spectral density with a peak off-resonant peak to molecular vibrational transitions (blue dashed line). (b) Cavity modification of chemical rate constant κ (κ_0 is the rate constant outside cavity) with unstructured (black) and structured (red) spectral density with a peak off-resonant peak showing that off-resonant peak in spectral density do not play a crucial role in cavity modification of chemical rate. Source data are provided as a Source Data file.

We investigate the role of off-resonant peaks in the spectral density for cavity-modified chemical reactivity. In the main text we showed two scenarios: (1) unstructured spectral density and (2) structured spectral density with peaks near molecular vibrational transitions. Here we demonstrate that off-resonant peaks in the spectral density (shown in Supplementary Fig. S13a) do not play crucial role for cavity modified chemical reaction rate. In Supplementary Fig. S13b we plot the cavity modified chemical reactivity for the unstructured (black dashed line), and compare it to a structured spectral density that includes a peak off-resonant with molecular transitions (red line). In both calculations we use the reaction coordinate potential used in the main text, consider a solvent friction of $\eta_s = 0.1\omega_b$, we consider a light-matter coupling to the reaction coordinate with a strength of $\eta_c = 0.0125$, and a cavity lifetime of $\tau_c = 1000$ fs. The off-resonant peak in the spectral density is included by coupling the reaction coordinate to spectator vibrational mode with frequency $\omega_Q = 1800$ cm-1 with a coupling constant of $C_Q = 2 \times 10^{-6}$, and a spectator solvent bath friction of $\lambda_s = 6.7 \times 10^{-7}$ a.u.. As can be seen, both curves are visually identical, demonstrating that off-resonant peak in the spectral density do not play a significant role for the cavity modified chemical rate.

Supplementary Note 9: Effect of light-matter coupling in suppressing chemical reactivity

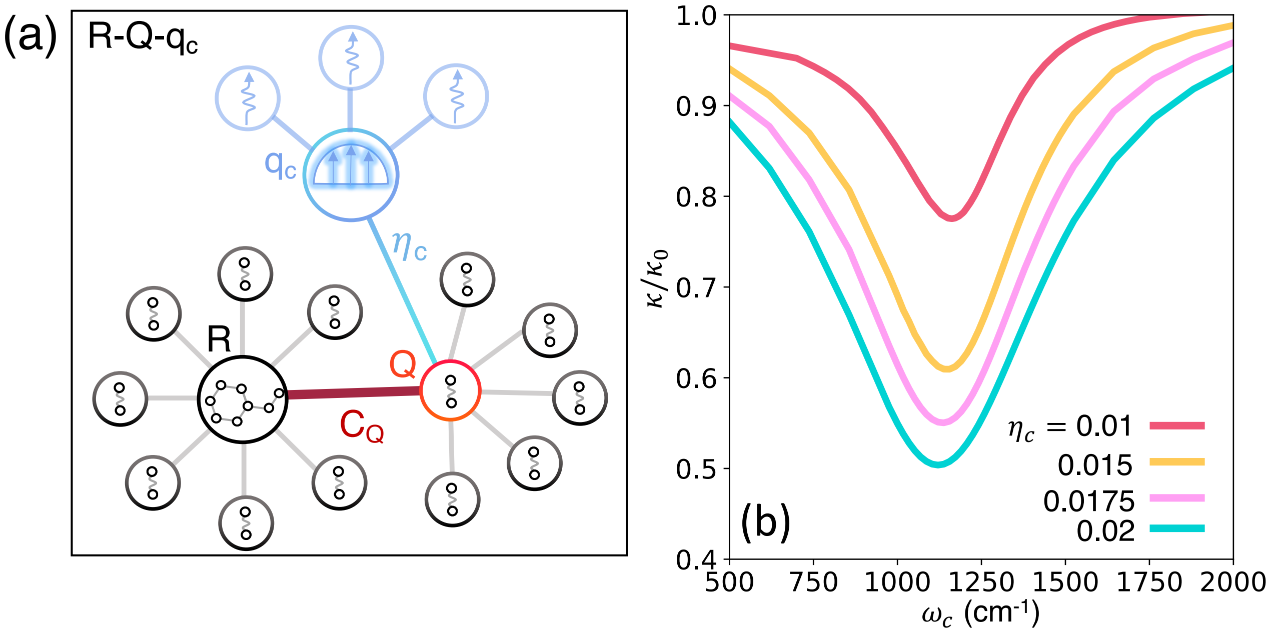

Supplementary Figure S14: **Suppression of chemical reactivity.** (a) Schematic diagram illustrating a solvent characterized by structured spectral density with the peak of the spectral density originating from a spectator mode with coordinate Q that is coupled to the cavity radiation mode with coordinate q_c. (b) Normalized chemical rate constant κ/κ_0 with κ_0 as chemical rate constant outside cavity as a function of cavity photon frequency at various light-matter coupling η_c. All parameters are same as in Fig. 5 in the main-text Source data are provided as a Source Data file.

We perform quantum dynamics simulations in a molecular system that is coupled to a structured spectral density where the peak of the spectral density originates from a spectator mode Q. Here we couple the spectator mode Q to a lossy cavity mode as illustrated in Supplementary Fig. S14a. We have varied the light-matter coupling strength η_c while keeping rest of the parameters the same as in Supplementary Fig. 5. As explained in the main-text, light-matter interactions between the Q mode and the cavity mode q_c shift the peak of the spectral density away from the molecular vibrational transition and lead to the suppression of the molecule-solvent interactions. This leads to the suppression of chemical reactivity. Supplementary Fig. S14b demonstrates that increasing light-matter coupling η_c further decreases the chemical rate, as expected.

Supplementary Note 10: Estimating quantization volume

To make a fair comparison to previous works [6, 7, 8, 9, 10, 11, 12], we estimated the quantization volume V by considering a proton transfer reaction, such that $\hat{\mu} = \frac{\hat{R}}{\sqrt{m_p}}$ (where $m_p = 1836$ a.u.). Thus, the $\eta_c \to \sqrt{m_p}\eta_c$ is scaled up when computing the quantization volume V. That is, V is estimated using the relation $\sqrt{m_p}\eta_c\omega_c = \sqrt{\hbar\omega_c/2\epsilon_0 V}$.

Supplementary References

[1] Shi, Q., Zhu, L. & Chen, L. Quantum rate dynamics for proton transfer reaction in a model system: Effect of the rate promoting vibrational mode. *J. Chem. Phys.* **135**, 044505 (2011).

[2] Pollak, E., Grabert, H. & Hänggi, P. Theory of activated rate processes for arbitrary frequency dependent friction: Solution of the turnover problem. *J. Chem. Phys.* **91**, 4073–4087 (1989).

[3] Li, T. E., Subotnik, J. E. & Nitzan, A. Cavity molecular dynamics simulations of liquid water under vibrational ultrastrong coupling. *Proc. Natl. Acad. Sci. U.S.A.* **117**, 18324–18331 (2020).

[4] Lindoy, L. P., Mandal, A. & Reichman, D. R. Resonant cavity modification of ground-state chemical kinetics. *J. Phys. Chem. Lett.* **13**, 6580–6586 (2022).

[5] Thomas, A. *et al.* Ground state chemistry under vibrational strong coupling: Dependence of thermodynamic parameters on the rabi splitting energy. *Nanophotonics* **9**, 249–255 (2020).

[6] Li, X., Mandal, A. & Huo, P. Cavity frequency-dependent theory for vibrational polariton chemistry. *Nat. Commun.* **12**, 1315 (2021).

[7] Li, X., Mandal, A. & Huo, P. Theory of mode-selective chemistry through polaritonic vibrational strong coupling. *J. Phys. Chem. Lett.* **12**, 6974–6982 (2021).

[8] Galego, J., Climent, C., Garcia-Vidal, F. J. & Feist, J. Cavity casimir-polder forces and their effects in ground-state chemical reactivity. *Phys. Rev. X* **9**, 021057 (2019).

[9] Sun, J. & Vendrell, O. Suppression and enhancement of thermal chemical rates in a cavity. *J. Phys. Chem. Lett.* **13**, 4441–4446 (2022).

[10] Philbin, J. P., Wang, Y., Narang, P. & Dou, W. Chemical reactions in imperfect cavities: Enhancement, suppression, and resonance. *J. Phys. Chem. C* **0**, null (2022).

[11] Schäfer, C., Flick, J., Ronca, E., Narang, P. & Rubio, A. Shining light on the microscopic resonant mechanism responsible for cavity-mediated chemical reactivity. *Nat. Commun.* **13**, 7817 (2022).

[12] Pavosevic, F., Hammes-Schiffer, S., Rubio, A. & Flick, J. Cavity-modulated proton transfer reactions. *J. Am. Chem. Soc.* **144**, 4995–5002 (2022).
