# Supplementary figures and images for: Quantum dynamical effects of vibrational strong coupling in chemical reactivity
Source: Nat Commun. 2023 May 12;14:2733. doi: 10.1038/s41467-023-38368-x (PMC10182063; doi:10.1038/s41467-023-38368-x)

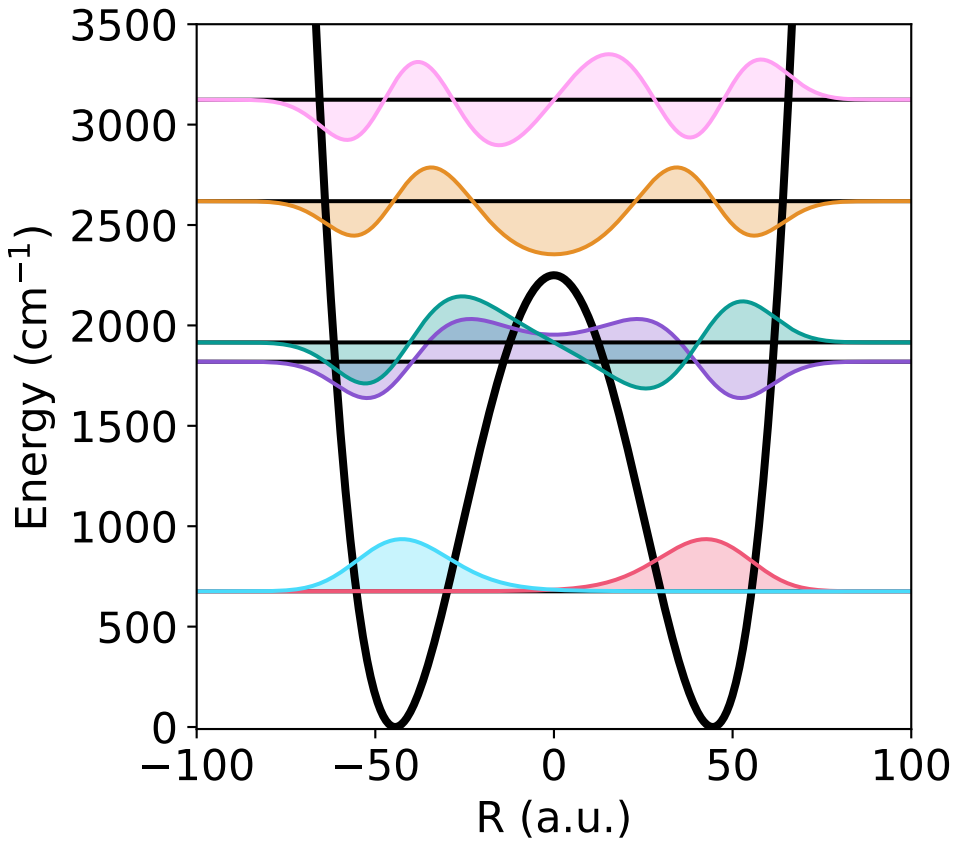

Supplement: Supplementary file 4 — Source Data [file 41467_2023_38368_MOESM4_ESM.zip › Data-VSC-HEOM-main/Fig1/fig1a.pdf]

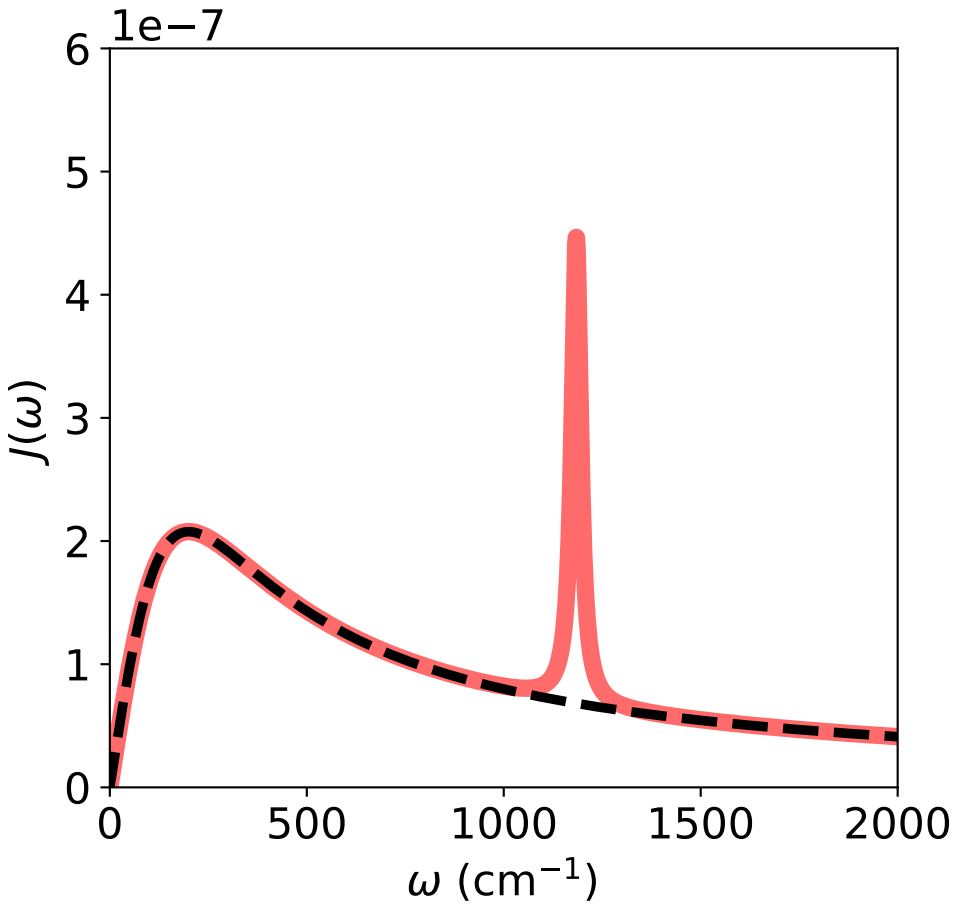

Supplement: Supplementary file 4 — Source Data [file 41467_2023_38368_MOESM4_ESM.zip › Data-VSC-HEOM-main/Fig1/fig1b.pdf]

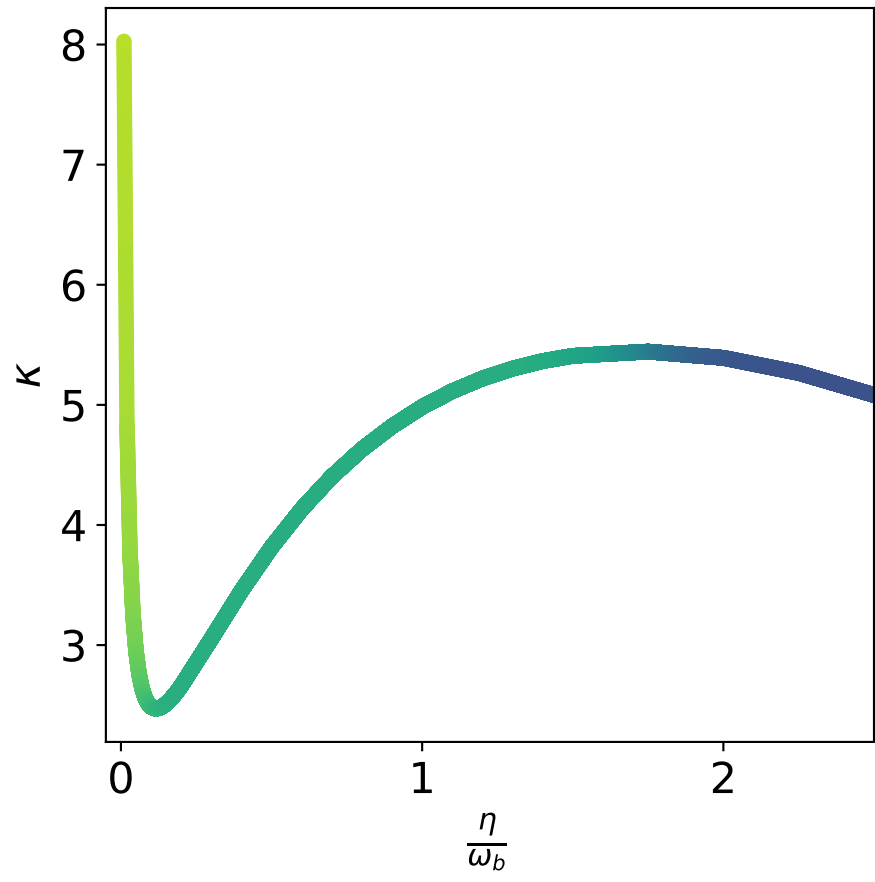

Supplement: Supplementary file 4 — Source Data [file 41467_2023_38368_MOESM4_ESM.zip › Data-VSC-HEOM-main/Fig1/fig1c.pdf]

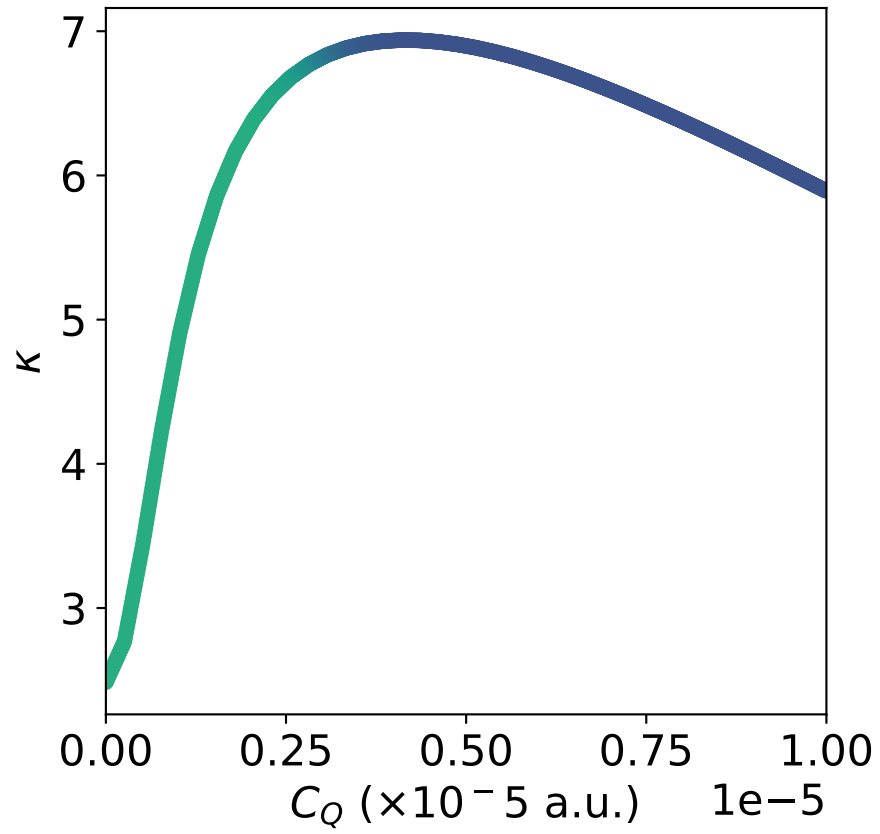

Supplement: Supplementary file 4 — Source Data [file 41467_2023_38368_MOESM4_ESM.zip › Data-VSC-HEOM-main/Fig1/fig1d.pdf]

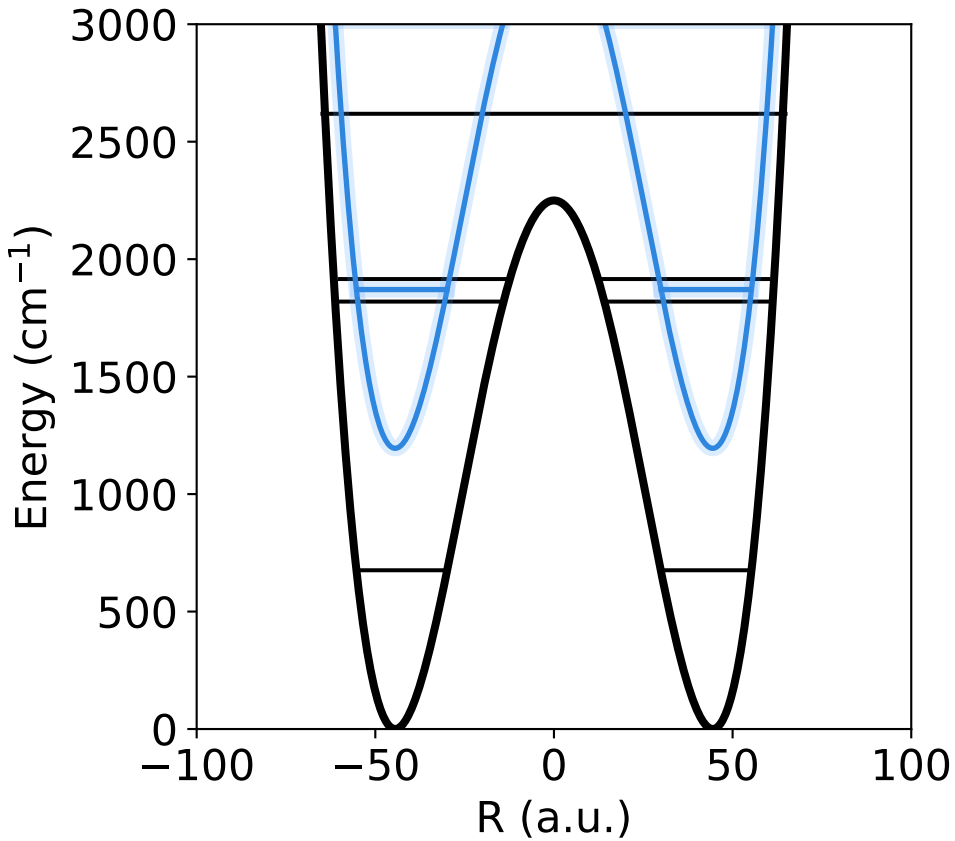

Supplement: Supplementary file 4 — Source Data [file 41467_2023_38368_MOESM4_ESM.zip › Data-VSC-HEOM-main/Fig2/fig2b.pdf]

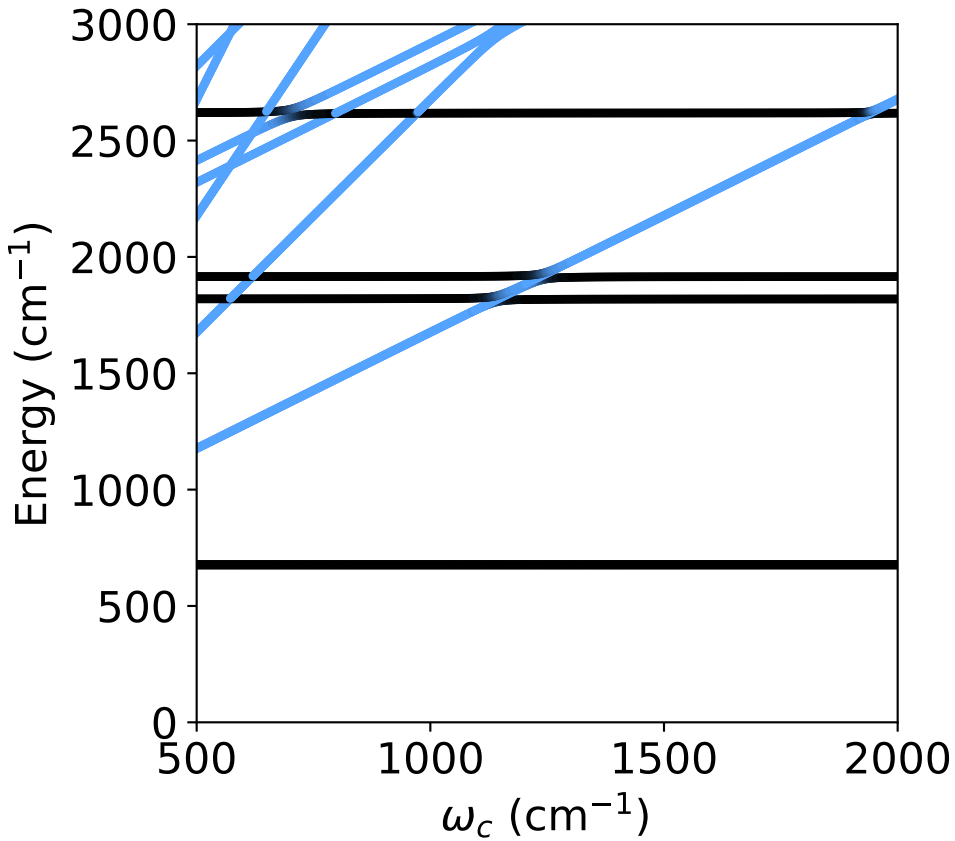

Supplement: Supplementary file 4 — Source Data [file 41467_2023_38368_MOESM4_ESM.zip › Data-VSC-HEOM-main/Fig2/fig2c.pdf]

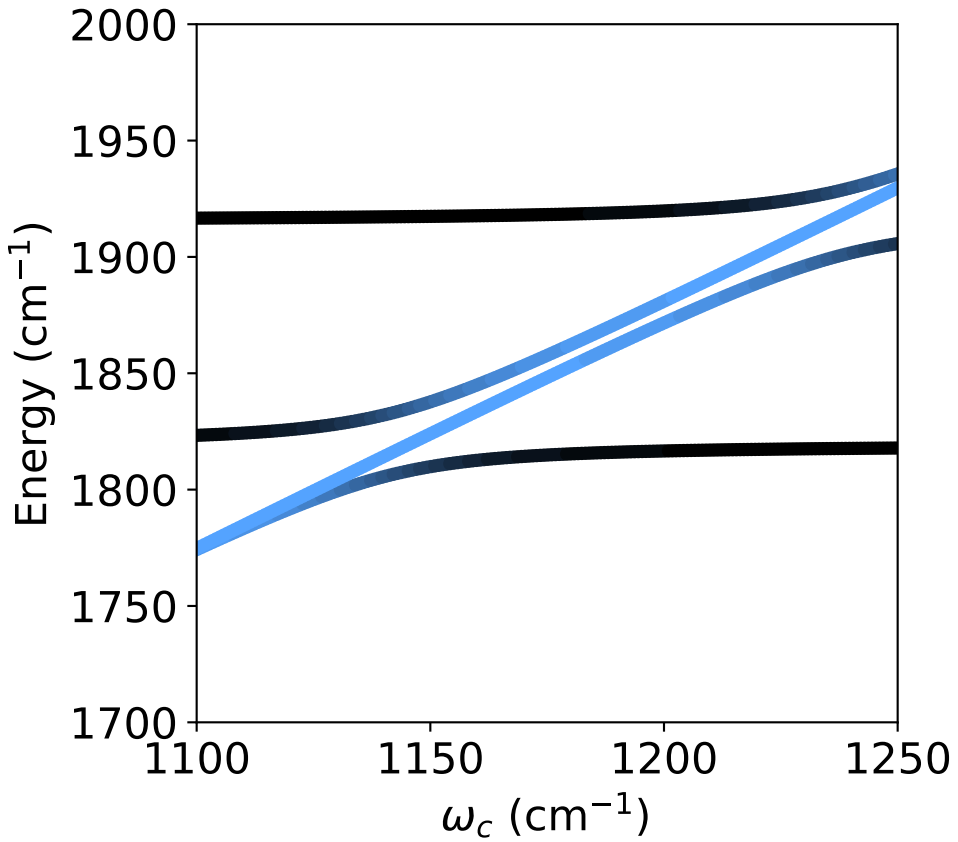

Supplement: Supplementary file 4 — Source Data [file 41467_2023_38368_MOESM4_ESM.zip › Data-VSC-HEOM-main/Fig2/fig2c_zoom.pdf]

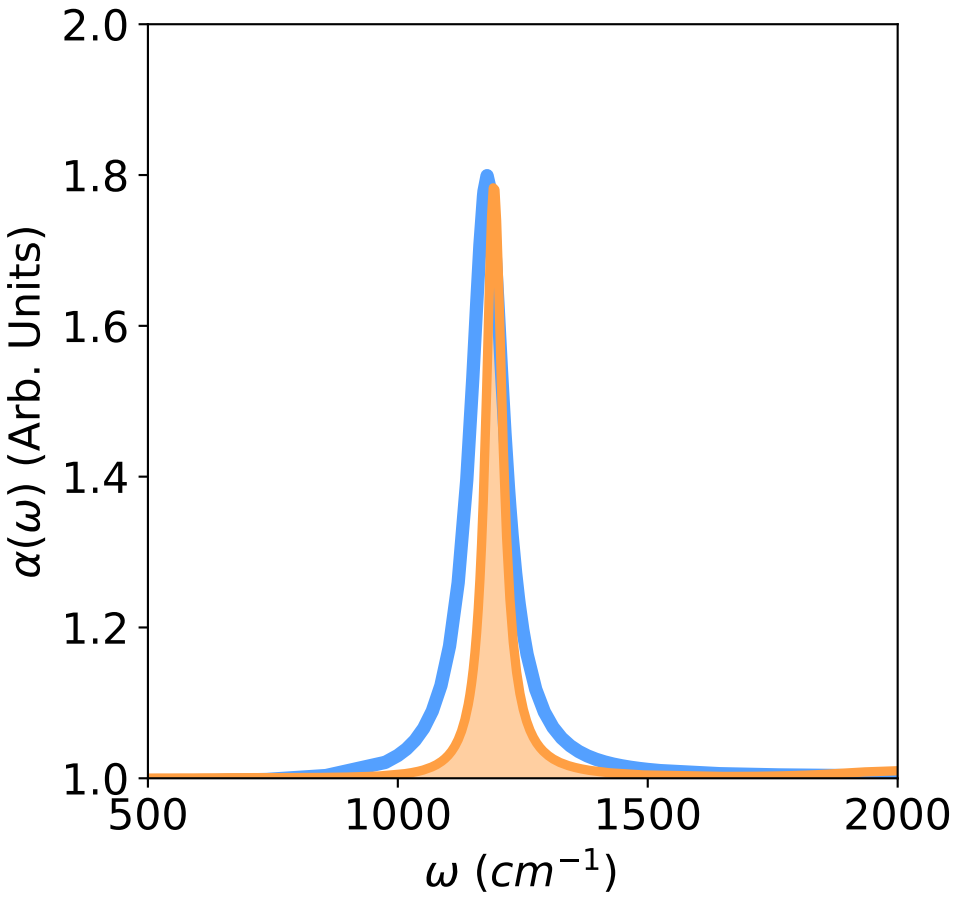

Supplement: Supplementary file 4 — Source Data [file 41467_2023_38368_MOESM4_ESM.zip › Data-VSC-HEOM-main/Fig2/fig2d.pdf]

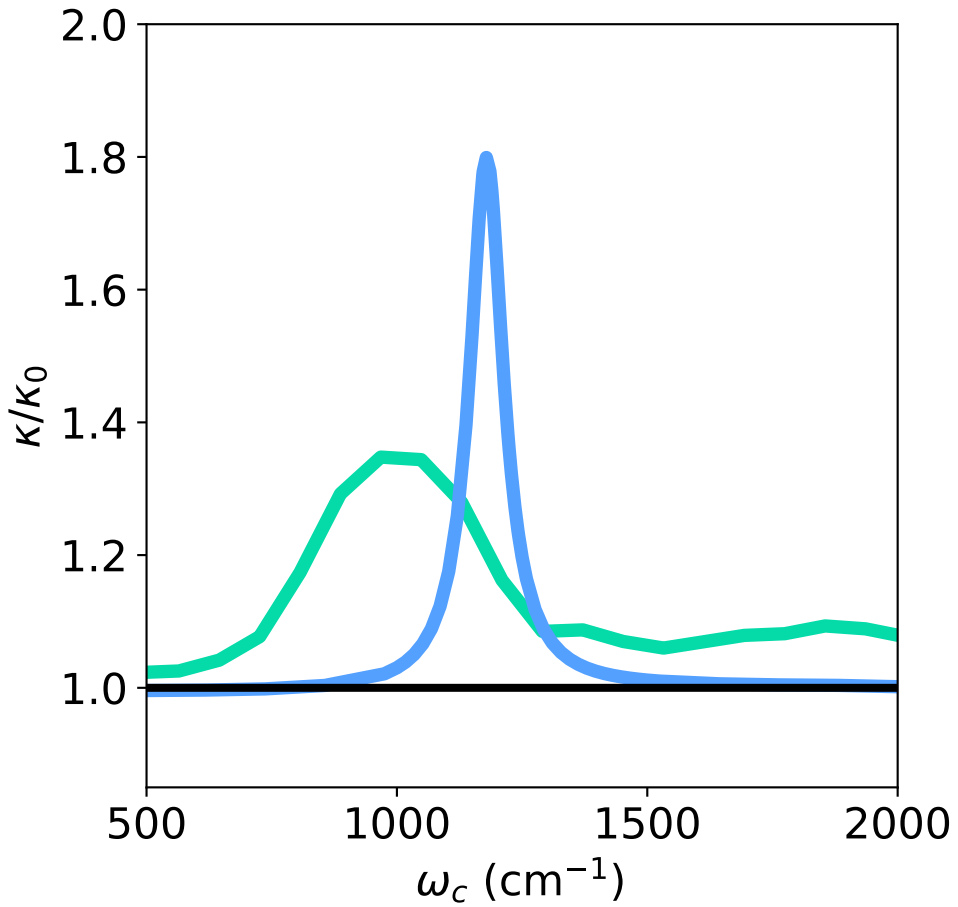

Supplement: Supplementary file 4 — Source Data [file 41467_2023_38368_MOESM4_ESM.zip › Data-VSC-HEOM-main/Fig2/fig2e.pdf]

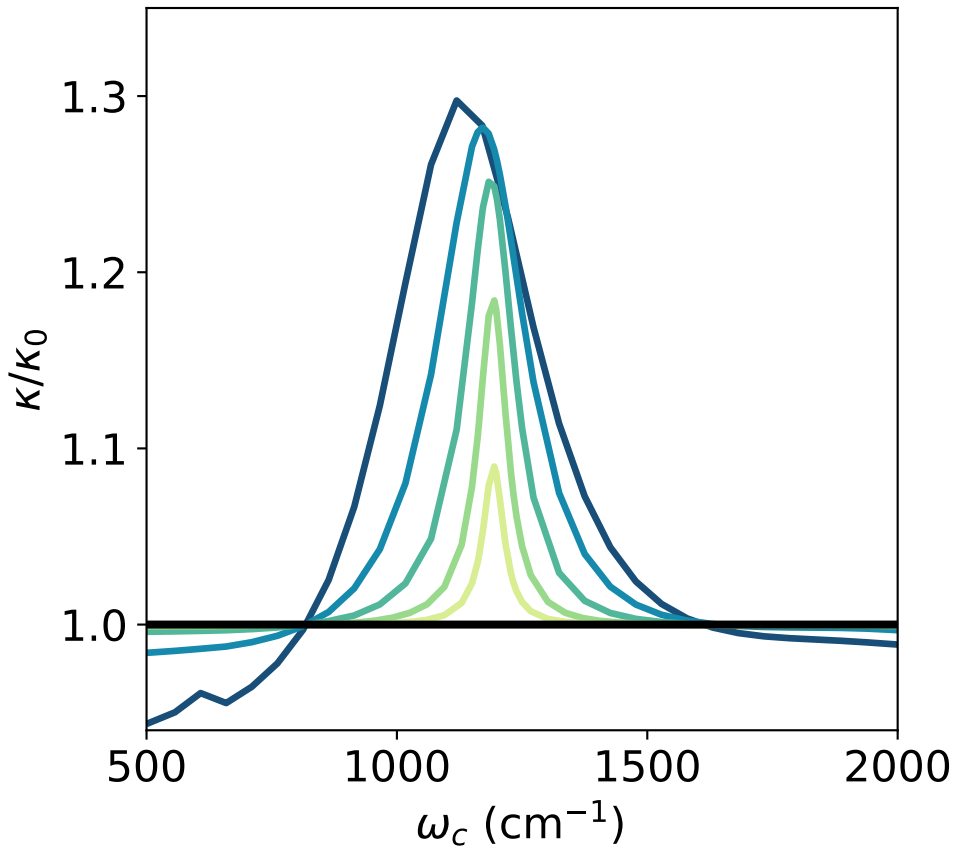

Supplement: Supplementary file 4 — Source Data [file 41467_2023_38368_MOESM4_ESM.zip › Data-VSC-HEOM-main/Fig3/fig3a.pdf]

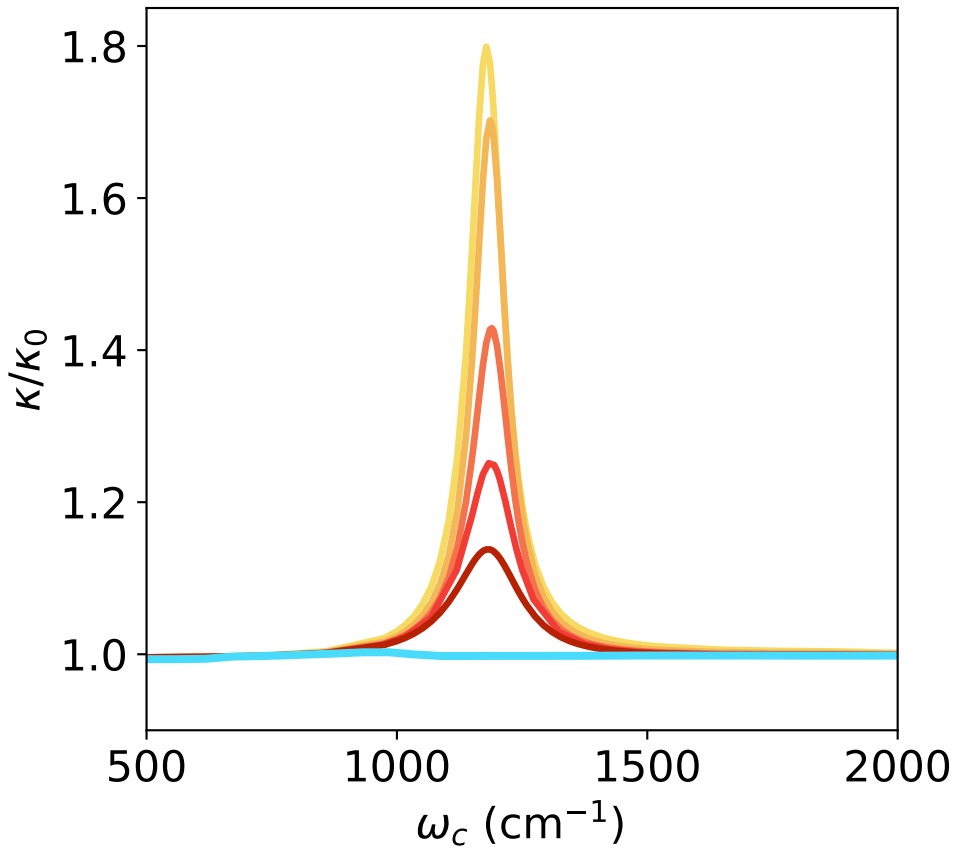

Supplement: Supplementary file 4 — Source Data [file 41467_2023_38368_MOESM4_ESM.zip › Data-VSC-HEOM-main/Fig3/fig3b.pdf]

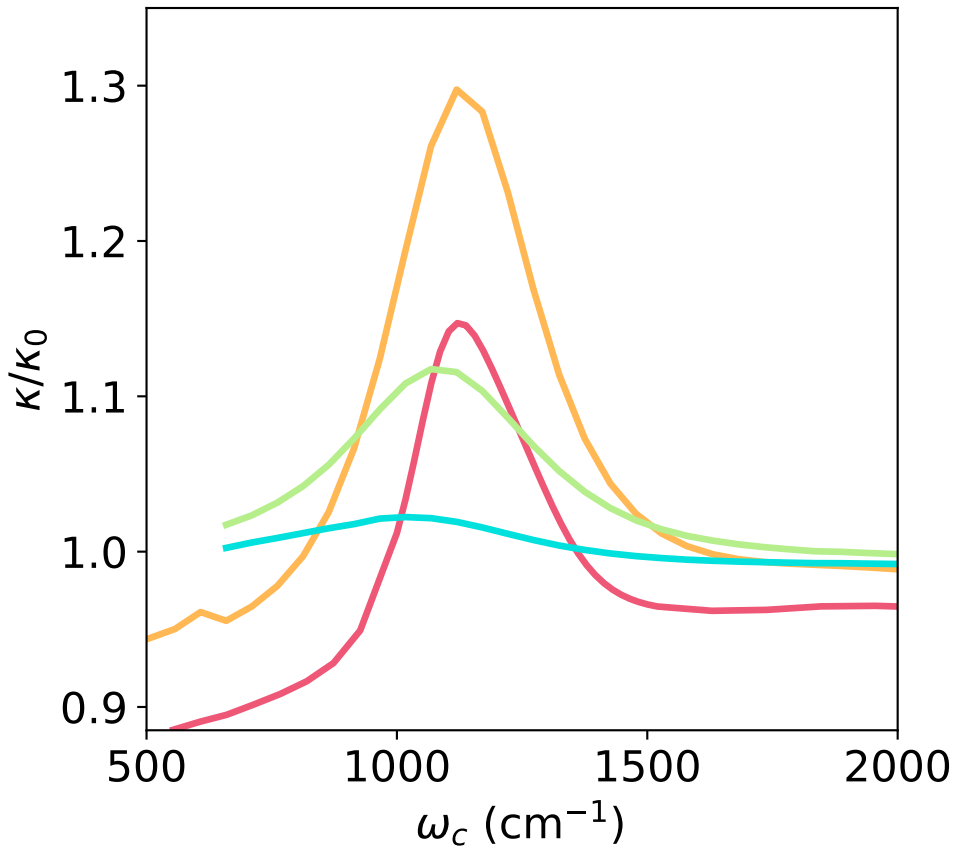

Supplement: Supplementary file 4 — Source Data [file 41467_2023_38368_MOESM4_ESM.zip › Data-VSC-HEOM-main/Fig3/fig3c.pdf]

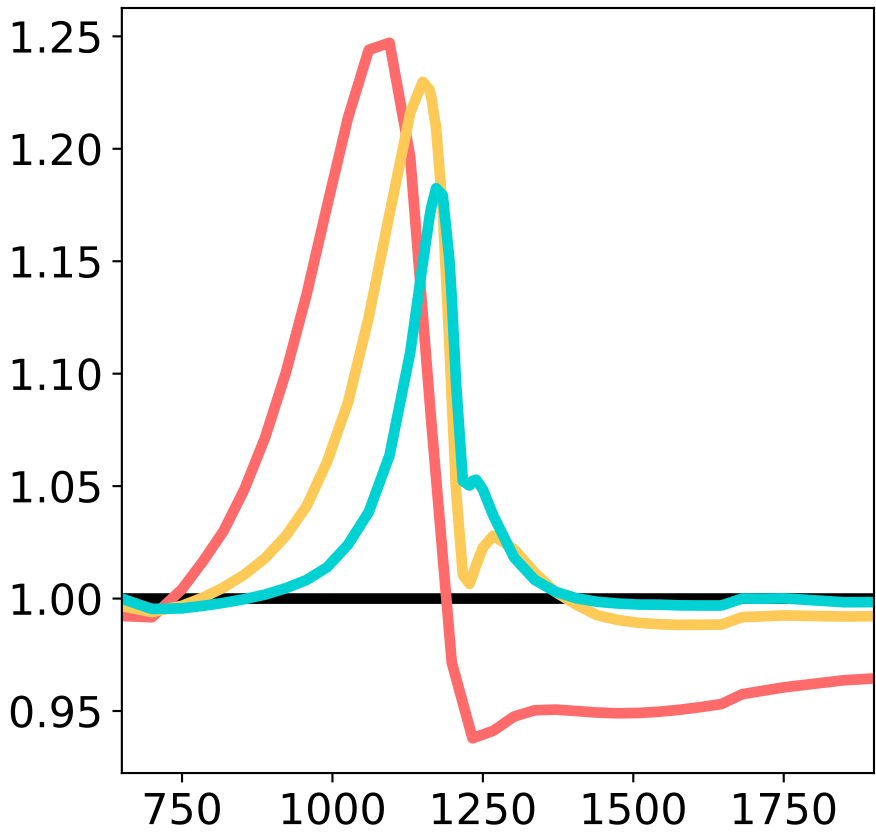

Supplement: Supplementary file 4 — Source Data [file 41467_2023_38368_MOESM4_ESM.zip › Data-VSC-HEOM-main/Fig4/fig4b.pdf]

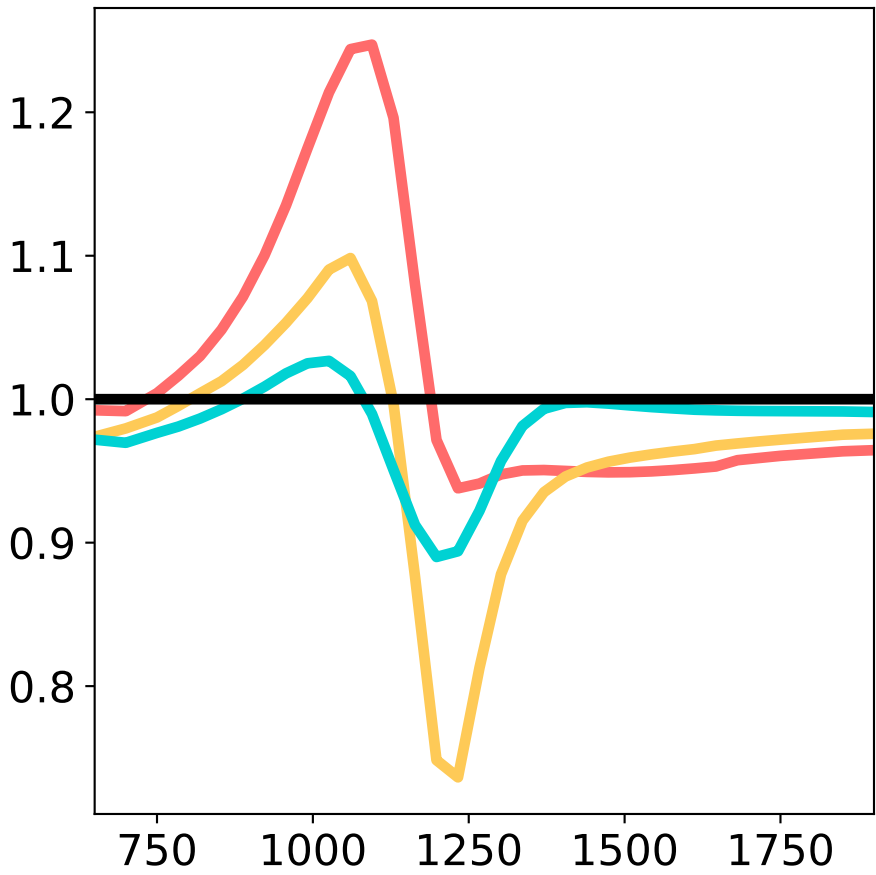

Supplement: Supplementary file 4 — Source Data [file 41467_2023_38368_MOESM4_ESM.zip › Data-VSC-HEOM-main/Fig4/fig4c.pdf]

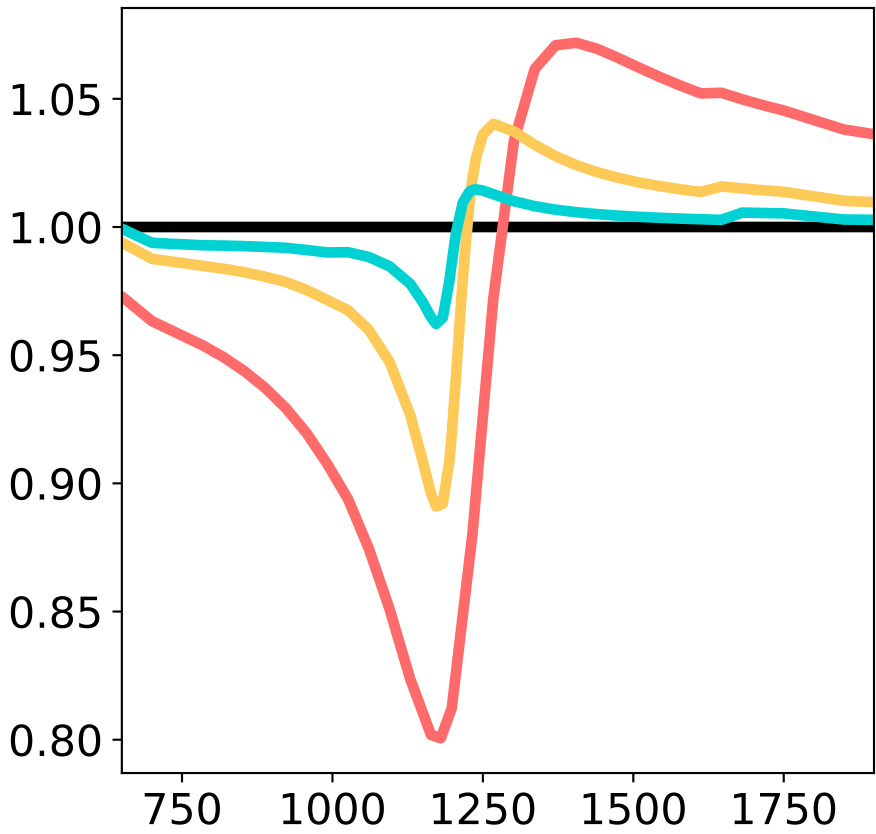

Supplement: Supplementary file 4 — Source Data [file 41467_2023_38368_MOESM4_ESM.zip › Data-VSC-HEOM-main/Fig4/fig4e.pdf]

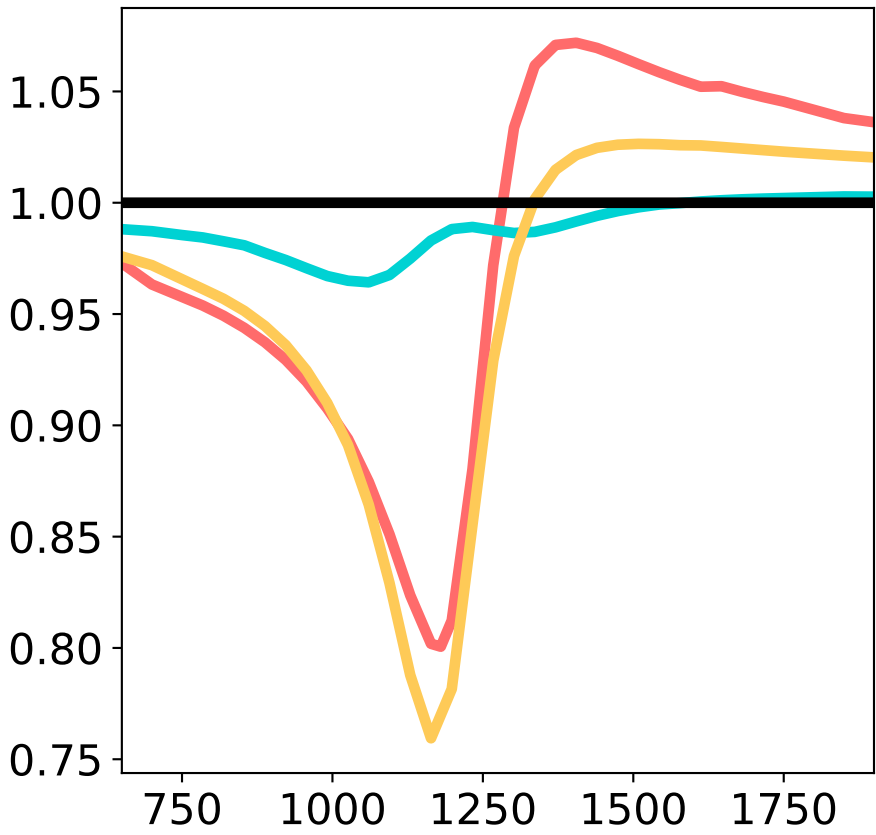

Supplement: Supplementary file 4 — Source Data [file 41467_2023_38368_MOESM4_ESM.zip › Data-VSC-HEOM-main/Fig4/fig4f.pdf]

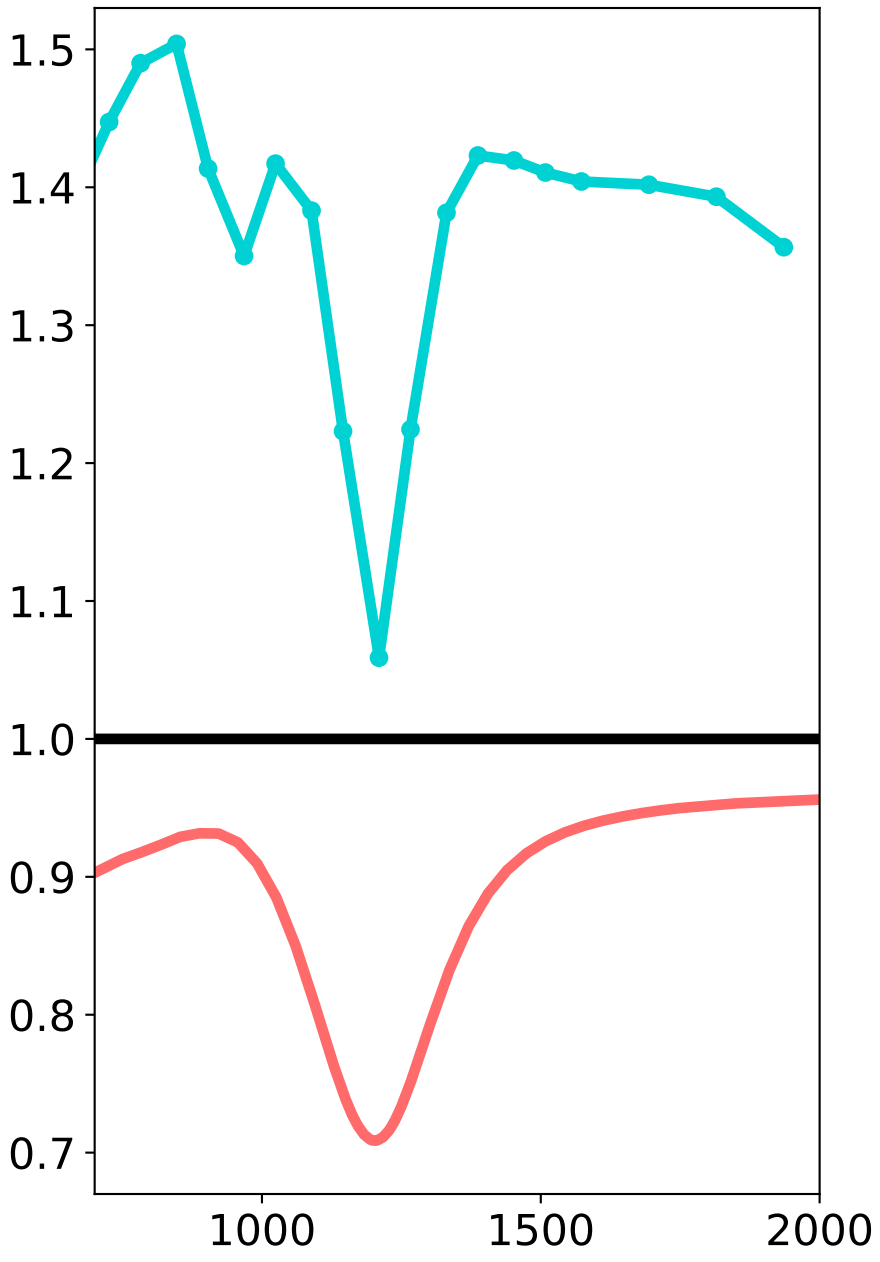

Supplement: Supplementary file 4 — Source Data [file 41467_2023_38368_MOESM4_ESM.zip › Data-VSC-HEOM-main/Fig5/fig5a.pdf]

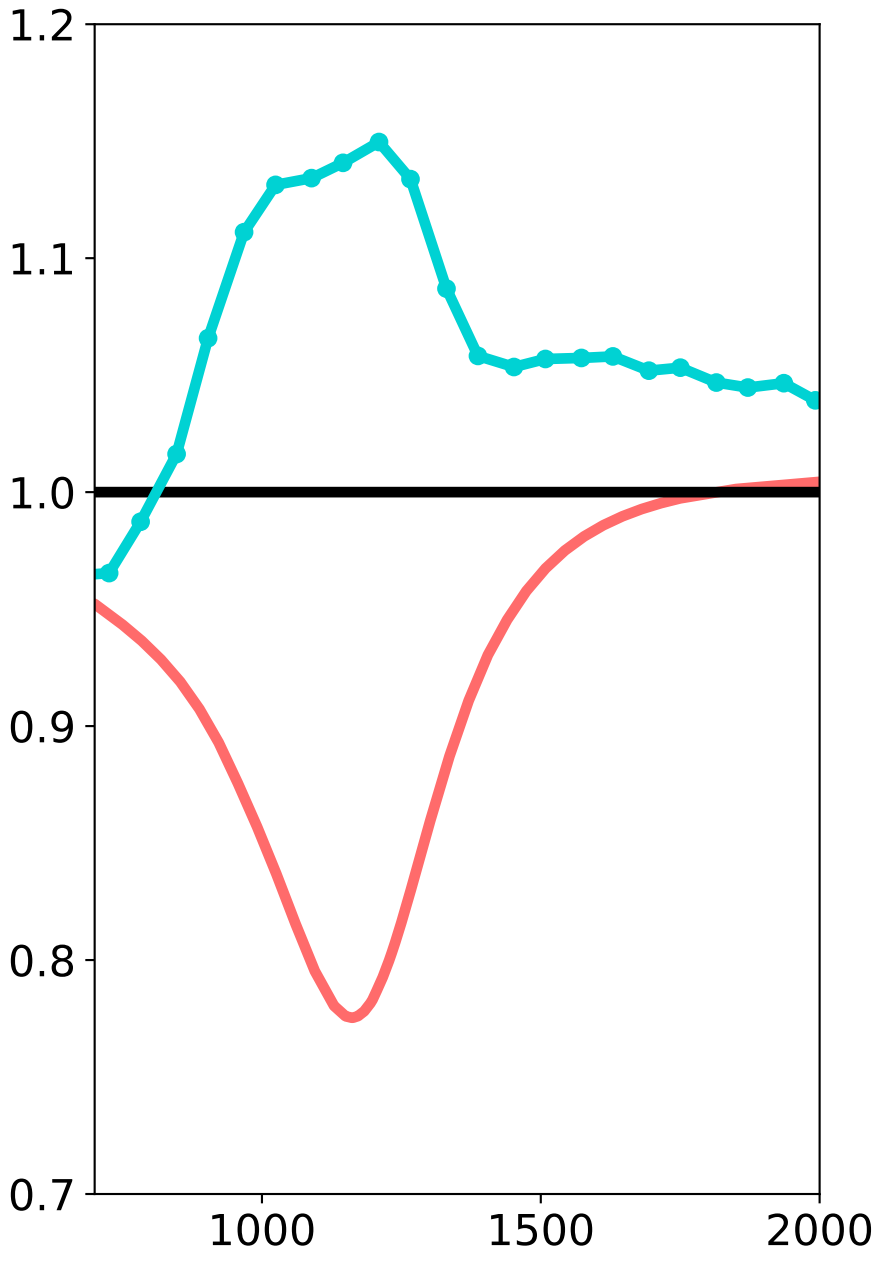

Supplement: Supplementary file 4 — Source Data [file 41467_2023_38368_MOESM4_ESM.zip › Data-VSC-HEOM-main/Fig5/fig5b.pdf]

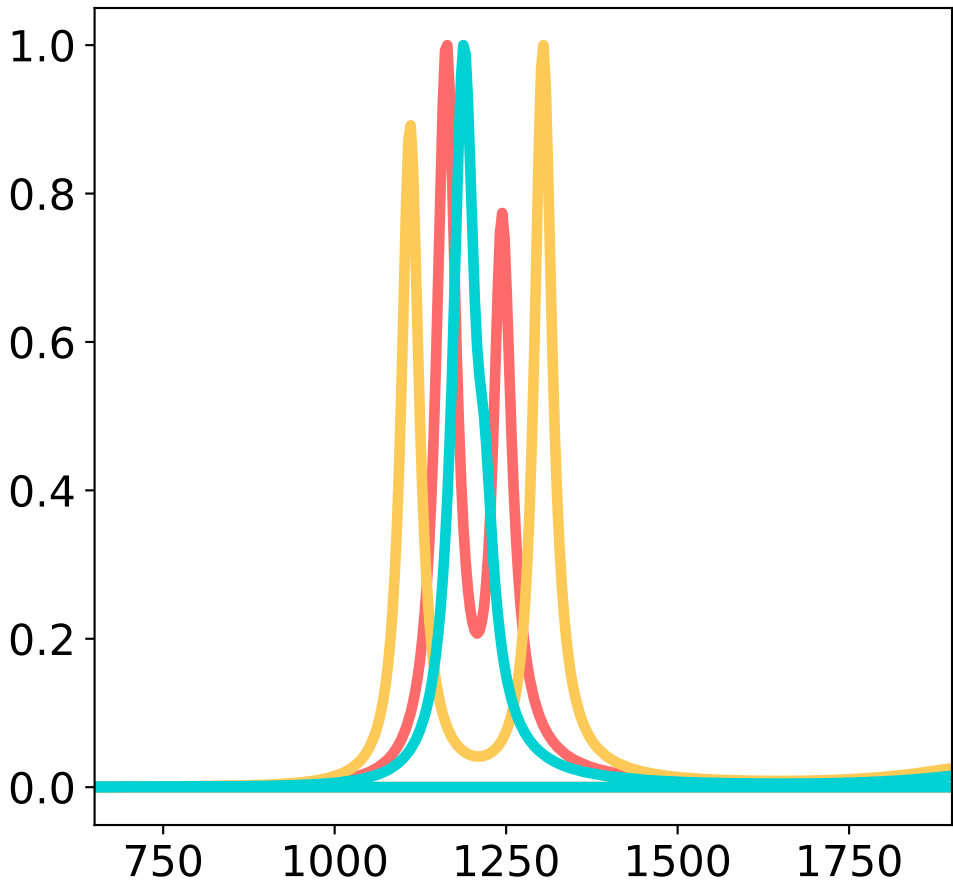

Supplement: Supplementary file 4 — Source Data [file 41467_2023_38368_MOESM4_ESM.zip › Data-VSC-HEOM-main/S1/S1a.pdf]

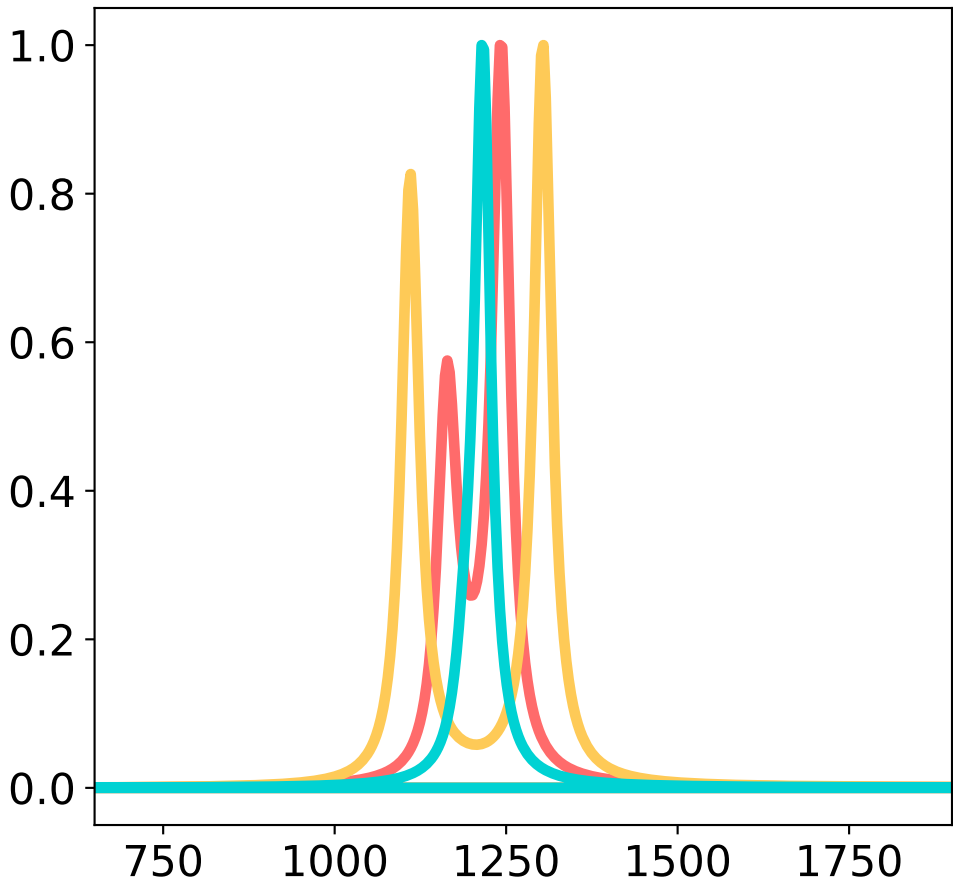

Supplement: Supplementary file 4 — Source Data [file 41467_2023_38368_MOESM4_ESM.zip › Data-VSC-HEOM-main/S1/S1b.pdf]

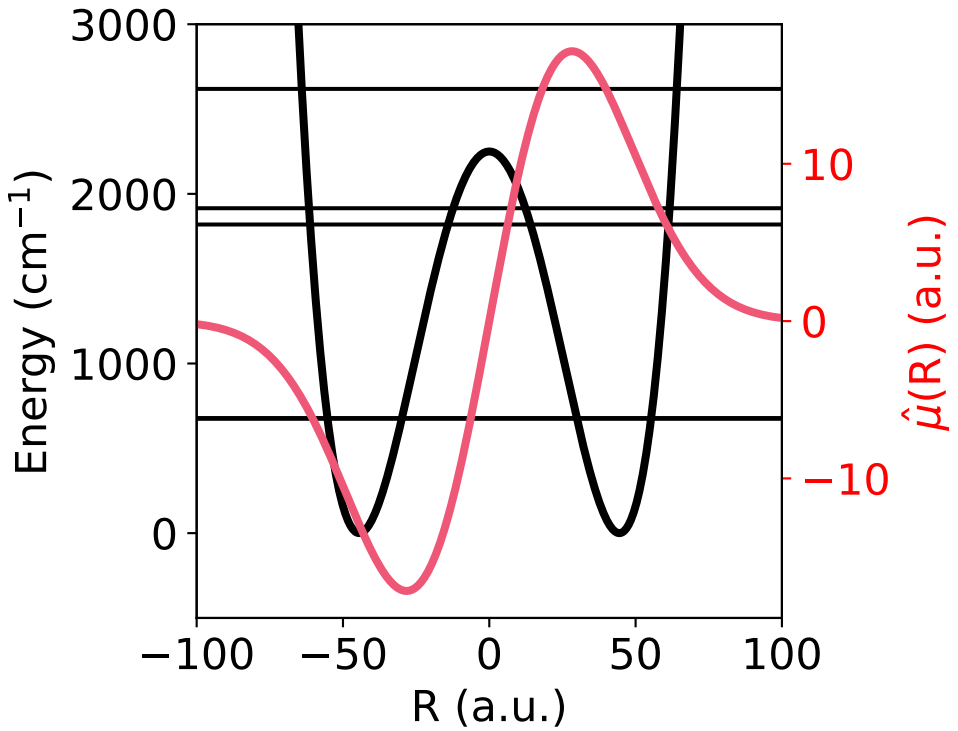

Supplement: Supplementary file 4 — Source Data [file 41467_2023_38368_MOESM4_ESM.zip › Data-VSC-HEOM-main/S10/S10a.pdf]

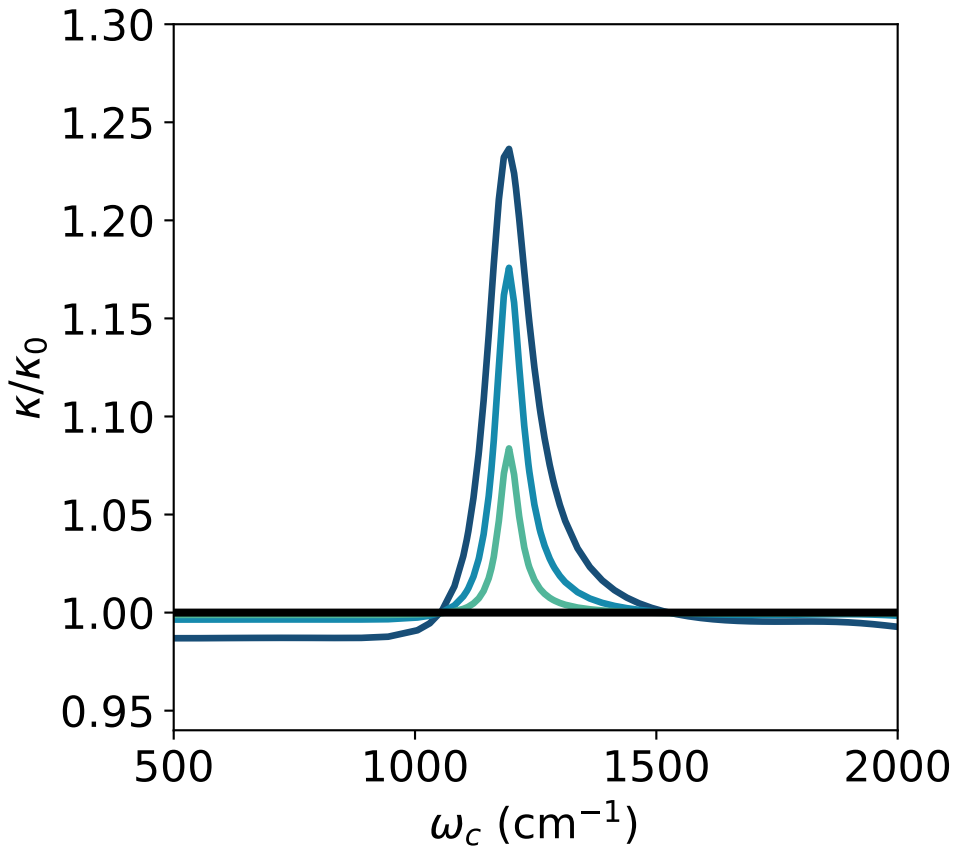

Supplement: Supplementary file 4 — Source Data [file 41467_2023_38368_MOESM4_ESM.zip › Data-VSC-HEOM-main/S10/S10b.pdf]

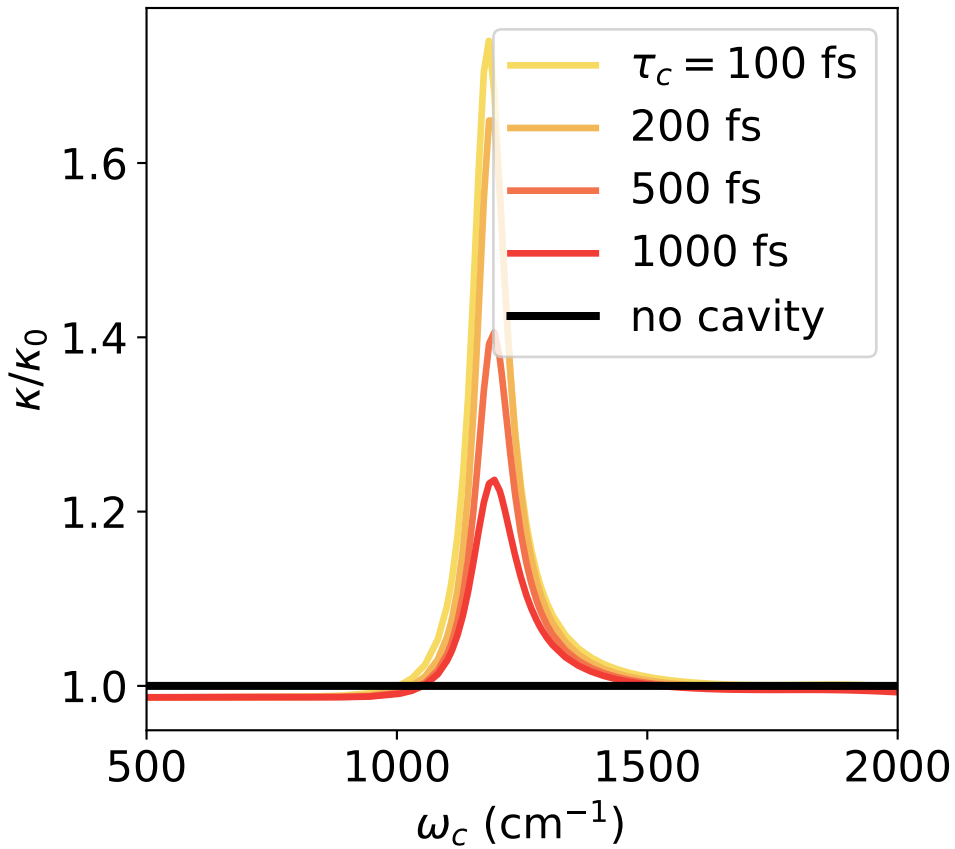

Supplement: Supplementary file 4 — Source Data [file 41467_2023_38368_MOESM4_ESM.zip › Data-VSC-HEOM-main/S10/S10c.pdf]

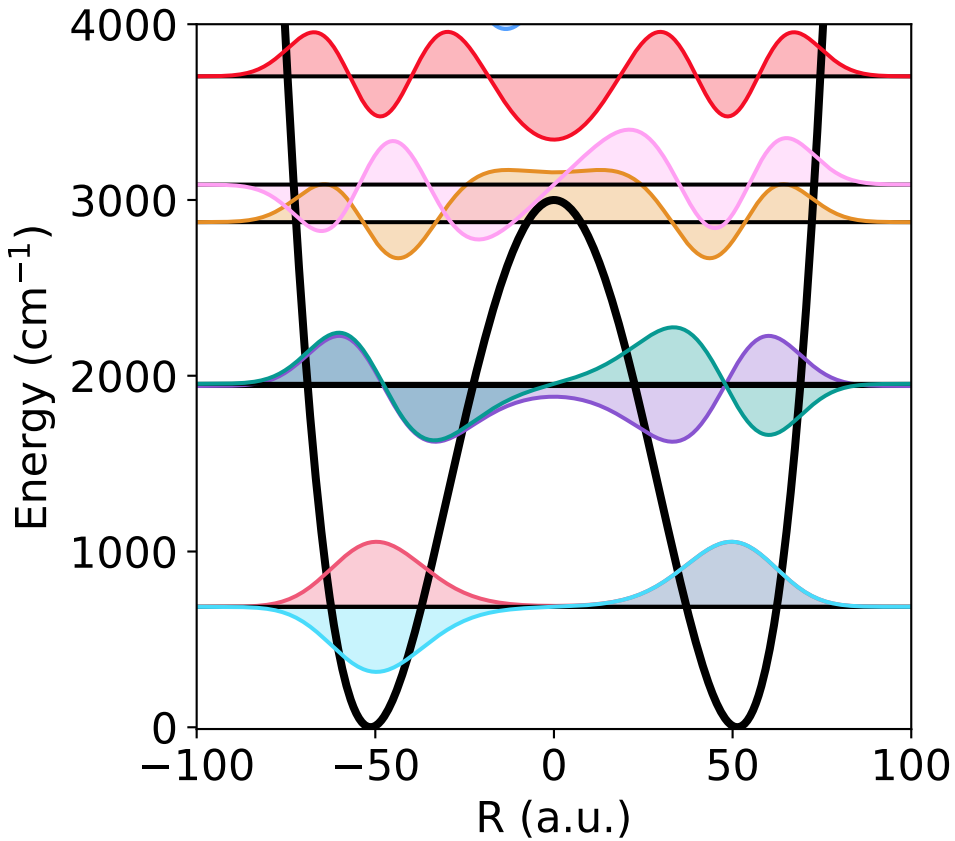

Supplement: Supplementary file 4 — Source Data [file 41467_2023_38368_MOESM4_ESM.zip › Data-VSC-HEOM-main/S11/S11a.pdf]

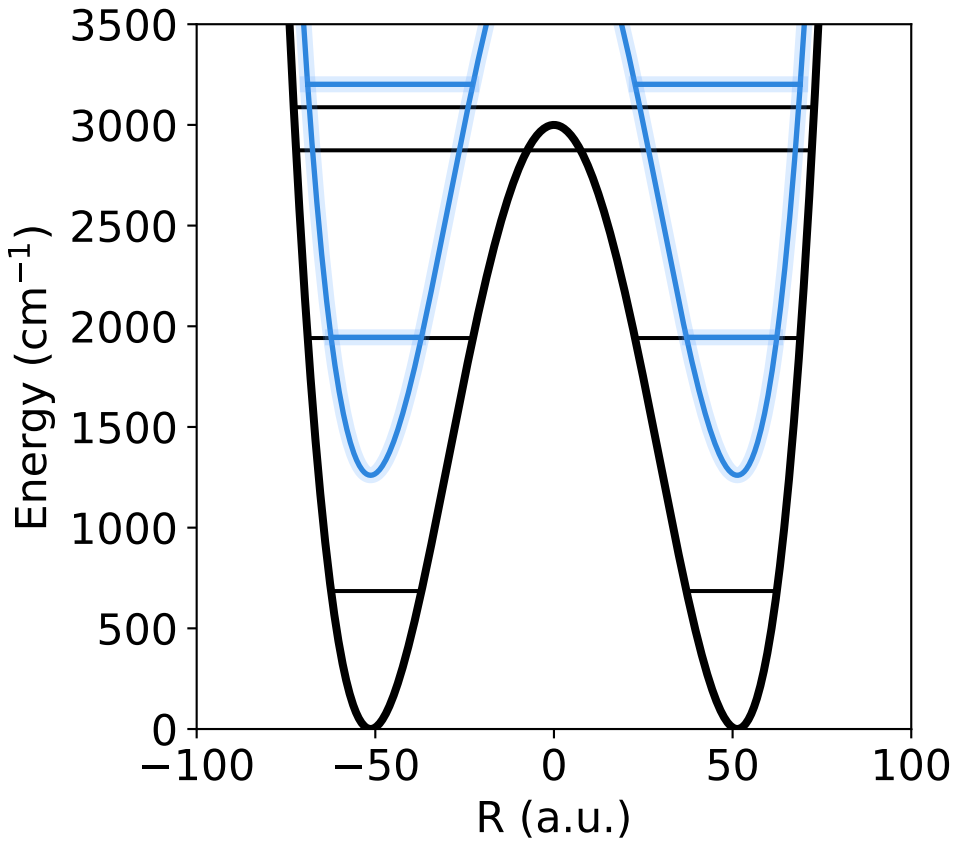

Supplement: Supplementary file 4 — Source Data [file 41467_2023_38368_MOESM4_ESM.zip › Data-VSC-HEOM-main/S11/S11b.pdf]

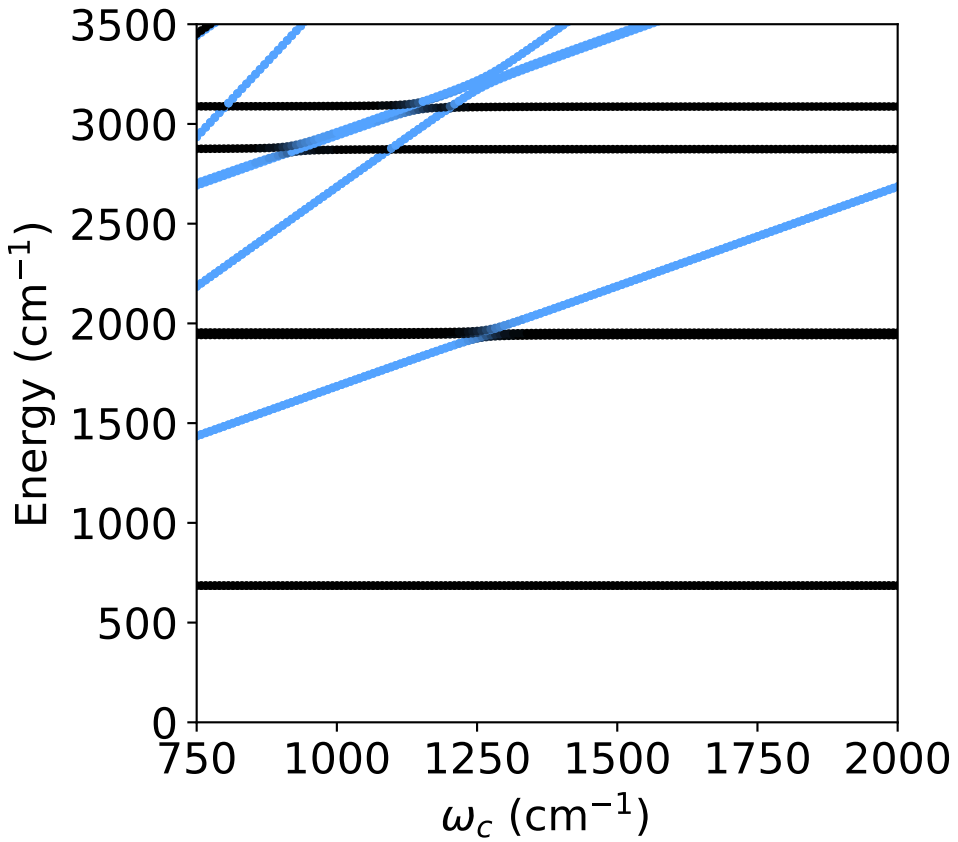

Supplement: Supplementary file 4 — Source Data [file 41467_2023_38368_MOESM4_ESM.zip › Data-VSC-HEOM-main/S11/S11c.pdf]

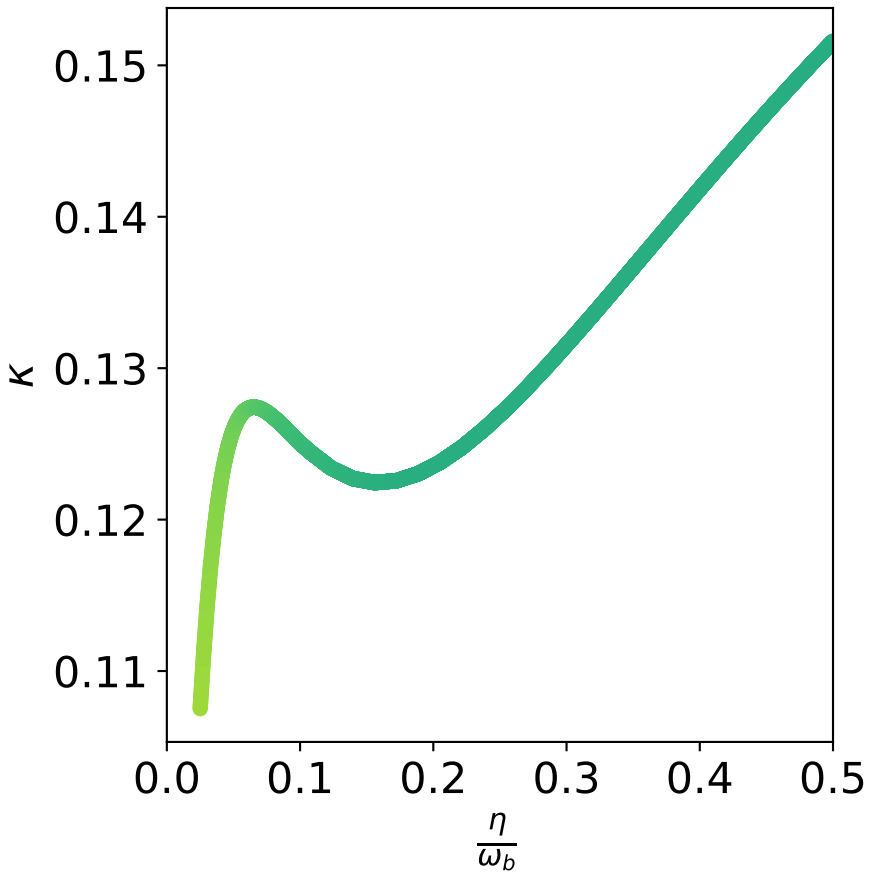

Supplement: Supplementary file 4 — Source Data [file 41467_2023_38368_MOESM4_ESM.zip › Data-VSC-HEOM-main/S11/S11d.pdf]

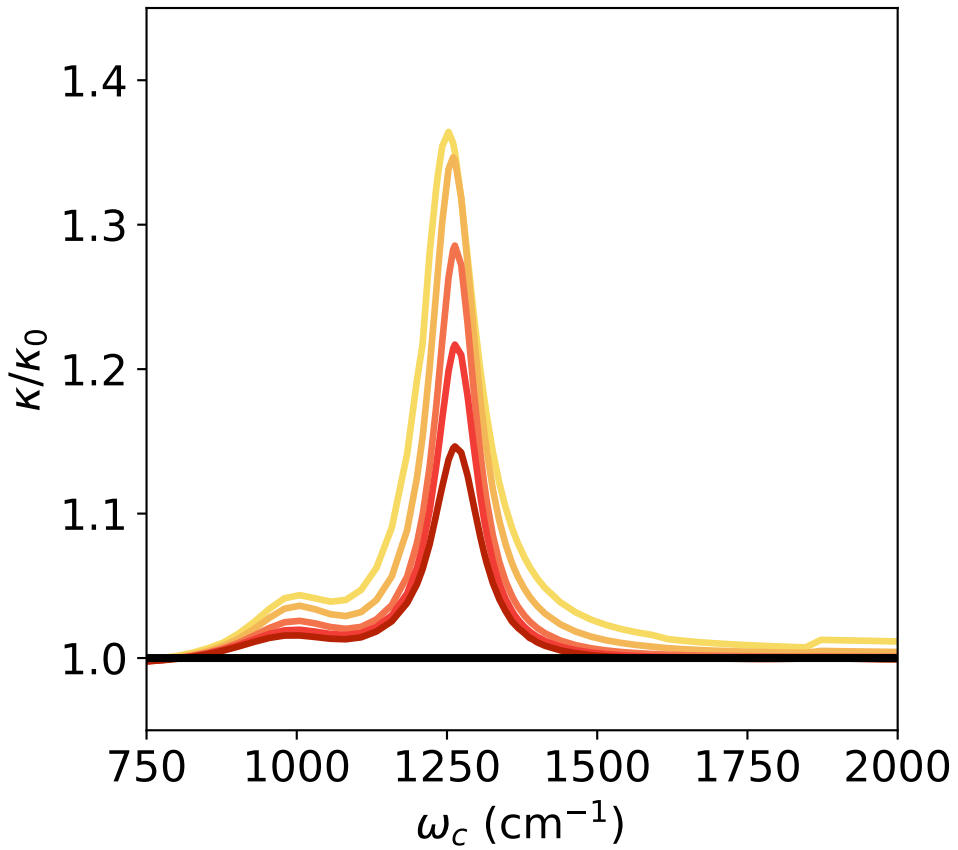

Supplement: Supplementary file 4 — Source Data [file 41467_2023_38368_MOESM4_ESM.zip › Data-VSC-HEOM-main/S11/S11e.pdf]

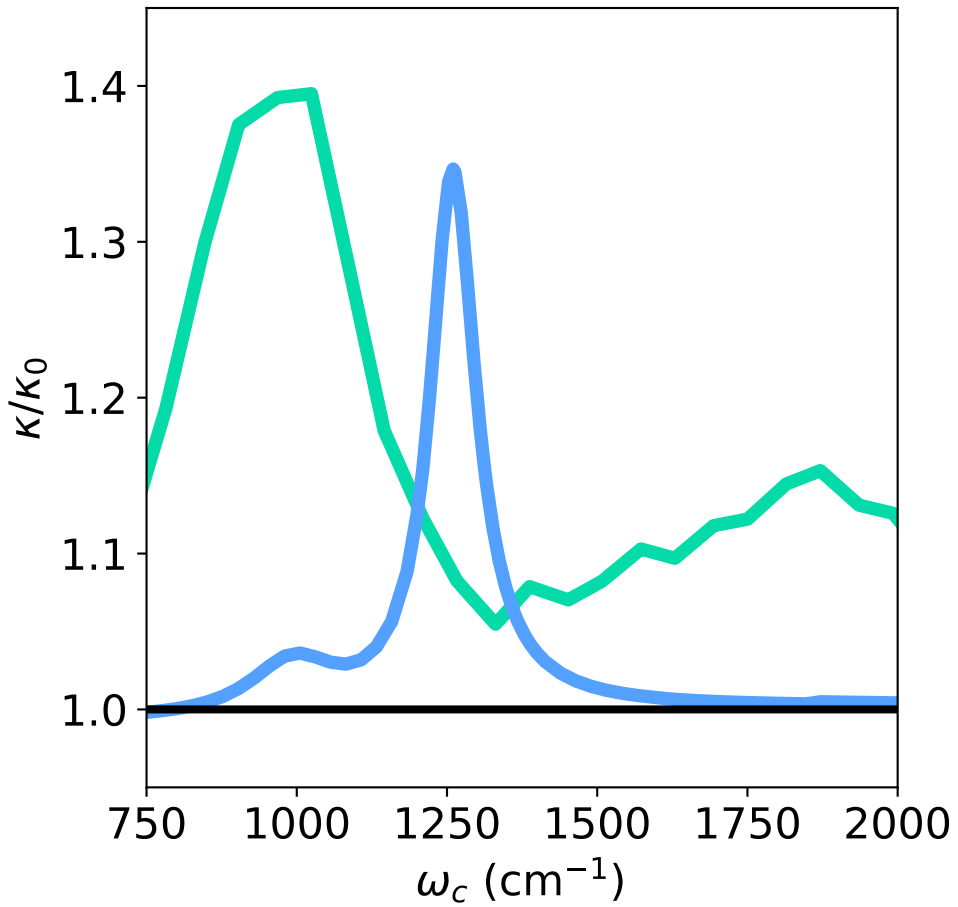

Supplement: Supplementary file 4 — Source Data [file 41467_2023_38368_MOESM4_ESM.zip › Data-VSC-HEOM-main/S11/S11f.pdf]

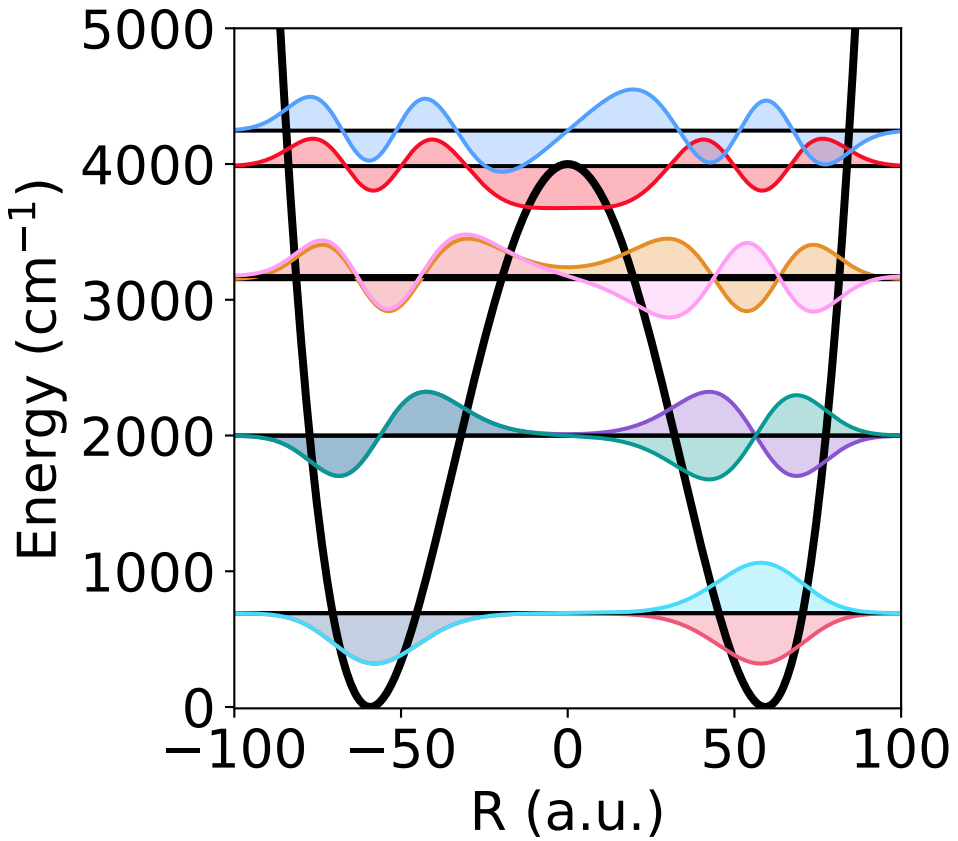

Supplement: Supplementary file 4 — Source Data [file 41467_2023_38368_MOESM4_ESM.zip › Data-VSC-HEOM-main/S12/S12a.pdf]

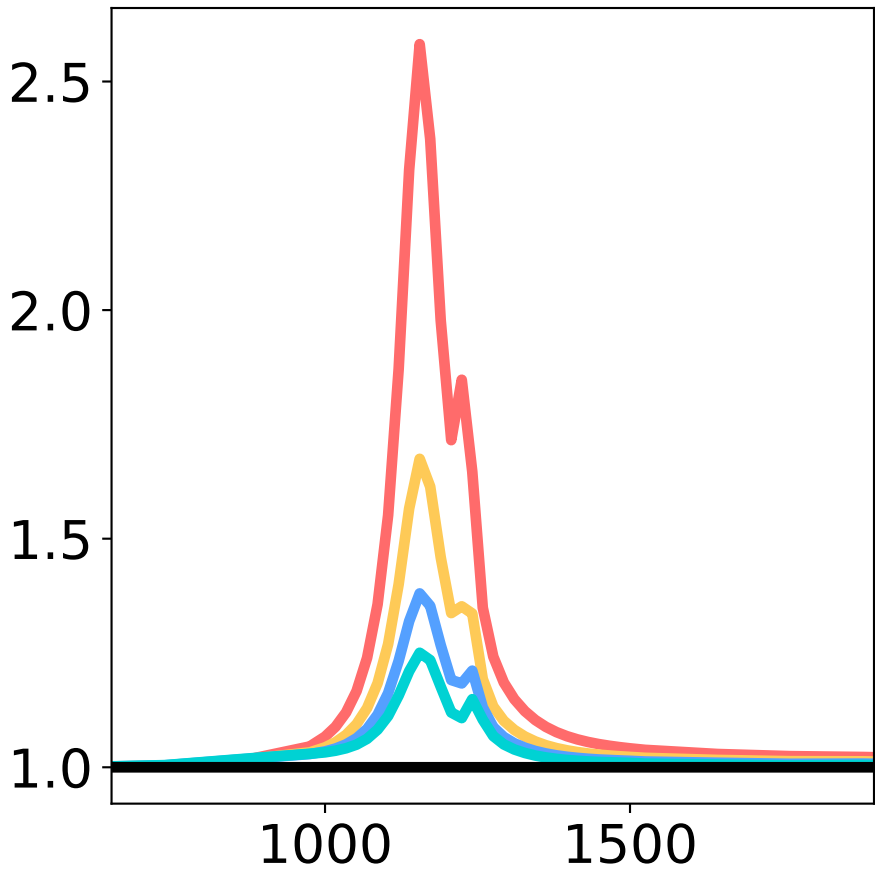

Supplement: Supplementary file 4 — Source Data [file 41467_2023_38368_MOESM4_ESM.zip › Data-VSC-HEOM-main/S12/S12b.pdf]

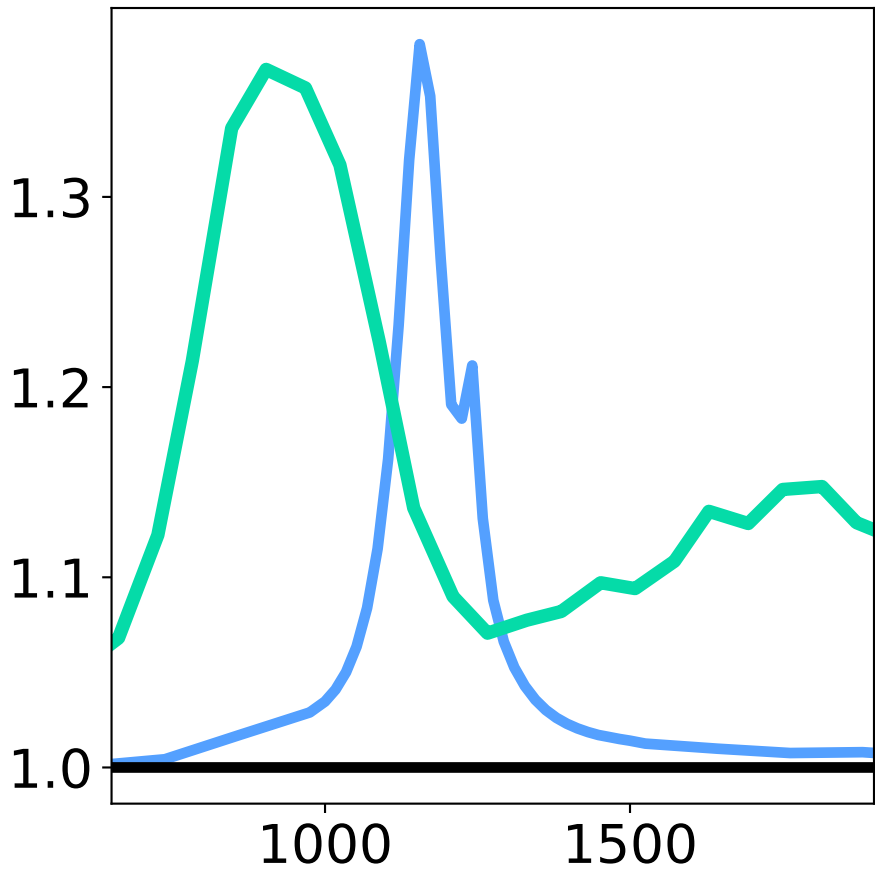

Supplement: Supplementary file 4 — Source Data [file 41467_2023_38368_MOESM4_ESM.zip › Data-VSC-HEOM-main/S12/S12c.pdf]

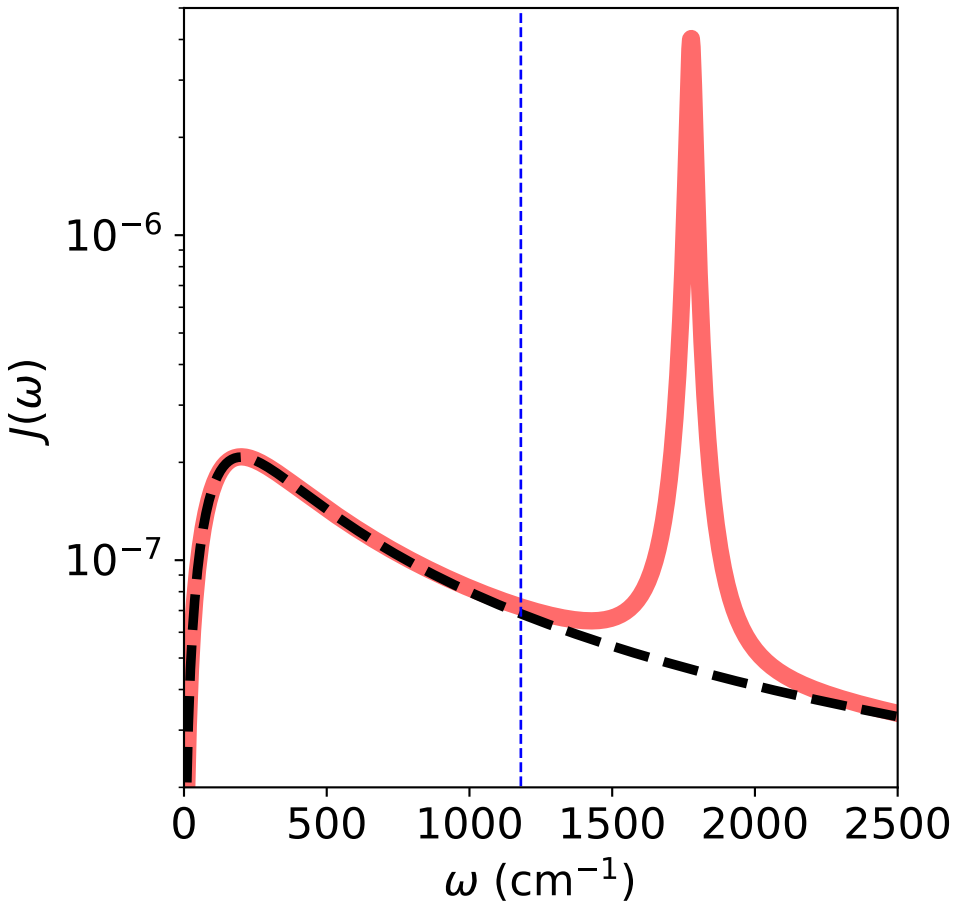

Supplement: Supplementary file 4 — Source Data [file 41467_2023_38368_MOESM4_ESM.zip › Data-VSC-HEOM-main/S13/S13a.pdf]

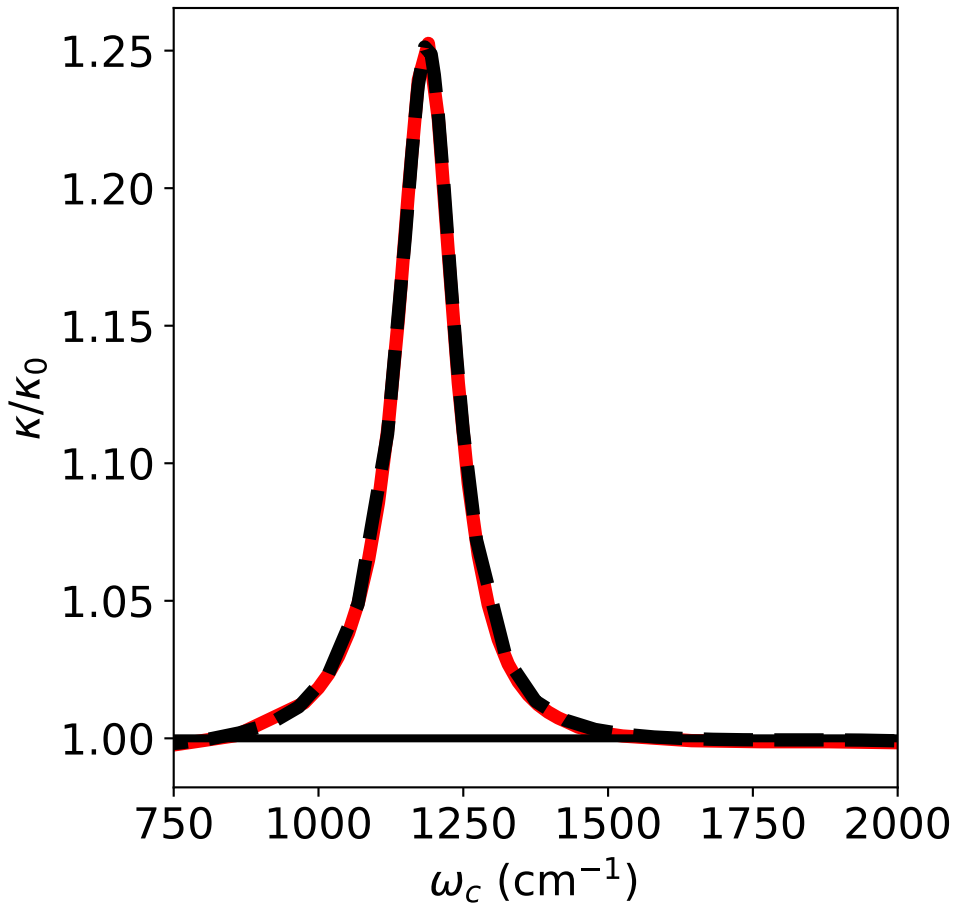

Supplement: Supplementary file 4 — Source Data [file 41467_2023_38368_MOESM4_ESM.zip › Data-VSC-HEOM-main/S13/S13b.pdf]

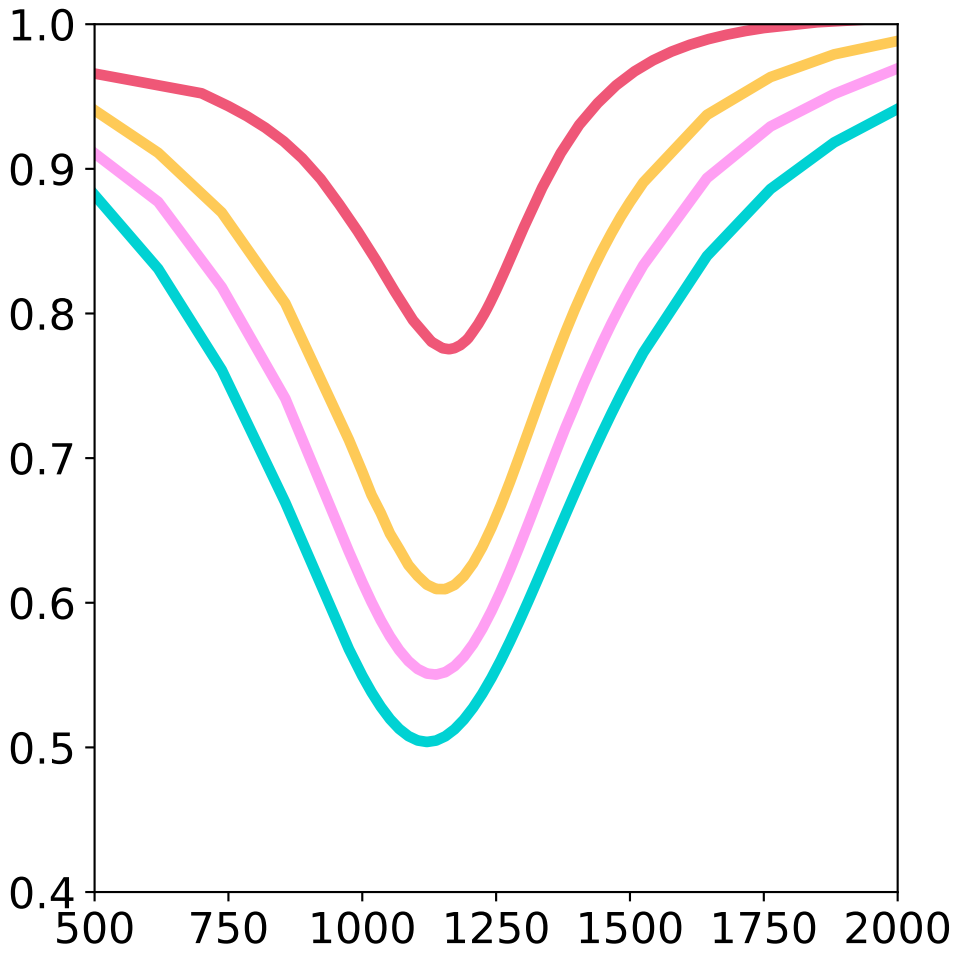

Supplement: Supplementary file 4 — Source Data [file 41467_2023_38368_MOESM4_ESM.zip › Data-VSC-HEOM-main/S14/S14b.pdf]

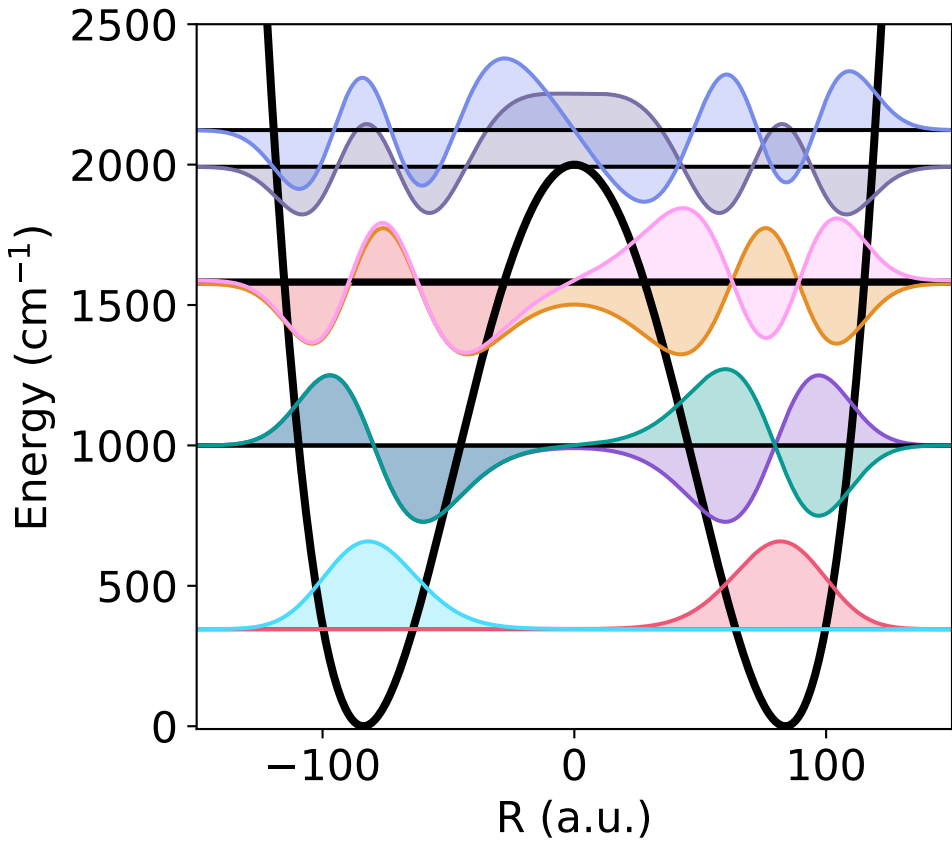

Supplement: Supplementary file 4 — Source Data [file 41467_2023_38368_MOESM4_ESM.zip › Data-VSC-HEOM-main/S2/S2a.pdf]

$1e-6$

$\kappa$

2.0

1.5

1.0

0.5

0.0

0.5

1.0

1.5

$\frac{\eta}{\omega_b}$

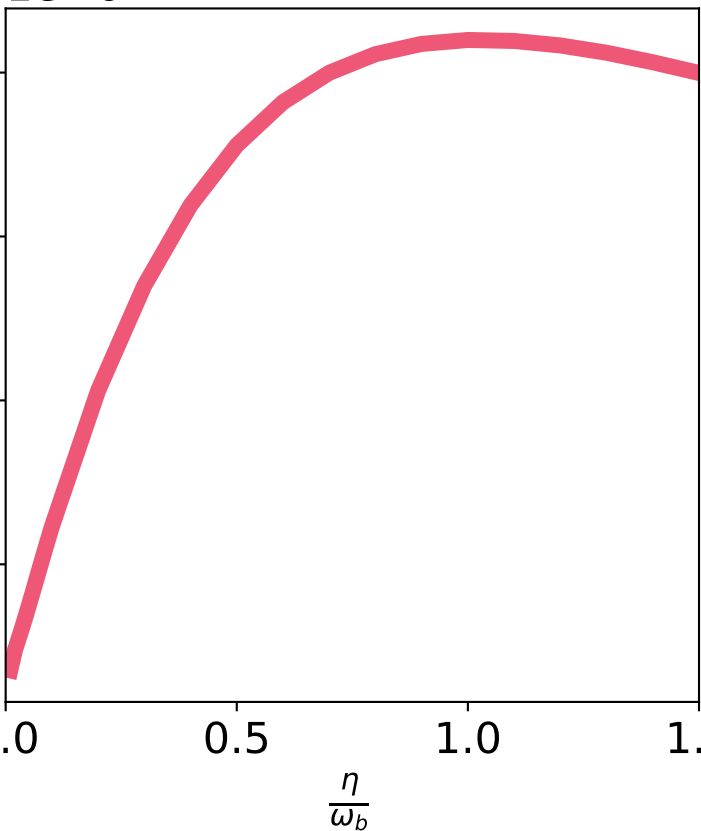

Supplement: Supplementary file 4 — Source Data [file 41467_2023_38368_MOESM4_ESM.zip › Data-VSC-HEOM-main/S2/S2b.pdf]

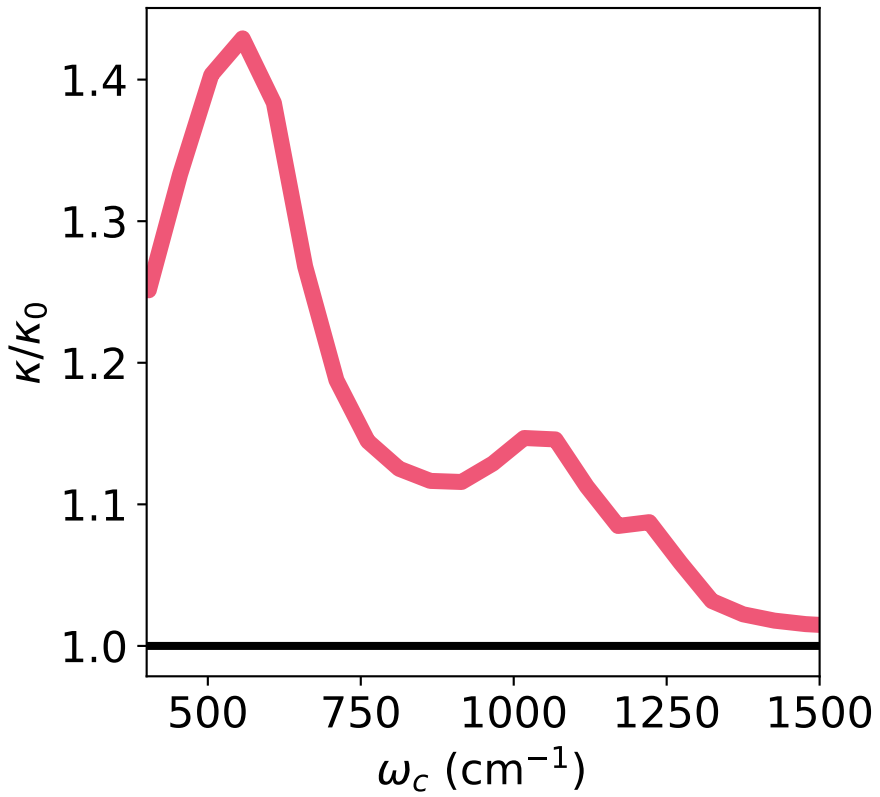

Supplement: Supplementary file 4 — Source Data [file 41467_2023_38368_MOESM4_ESM.zip › Data-VSC-HEOM-main/S2/S2c.pdf]

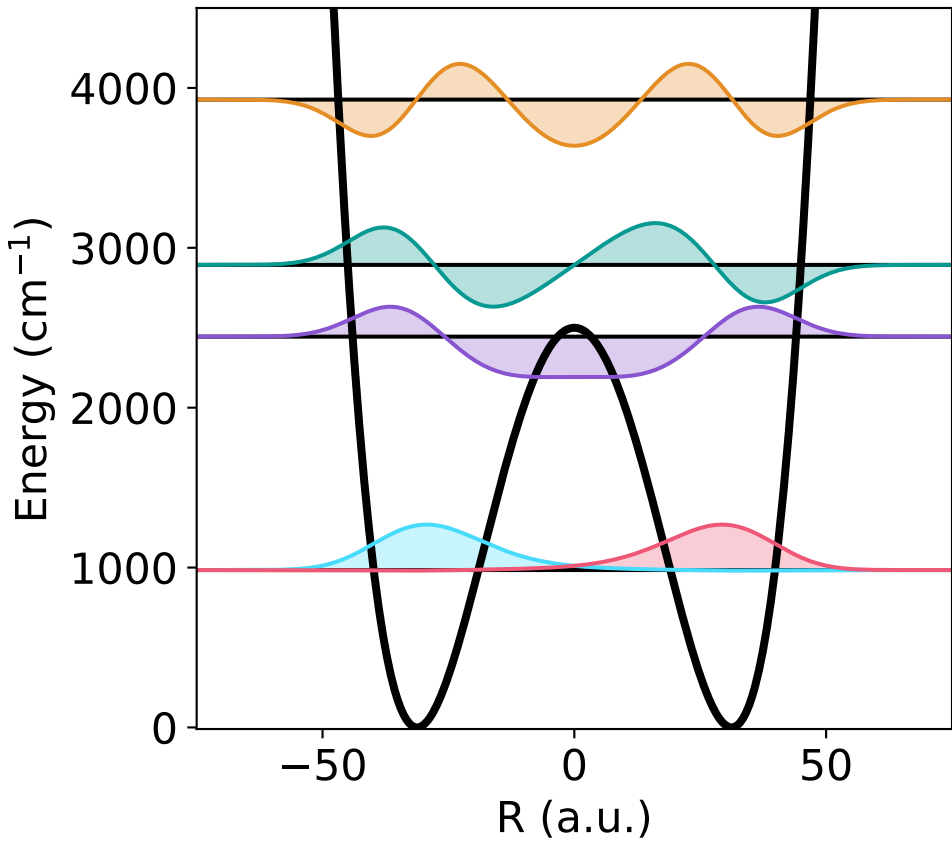

Supplement: Supplementary file 4 — Source Data [file 41467_2023_38368_MOESM4_ESM.zip › Data-VSC-HEOM-main/S3/S3a.pdf]

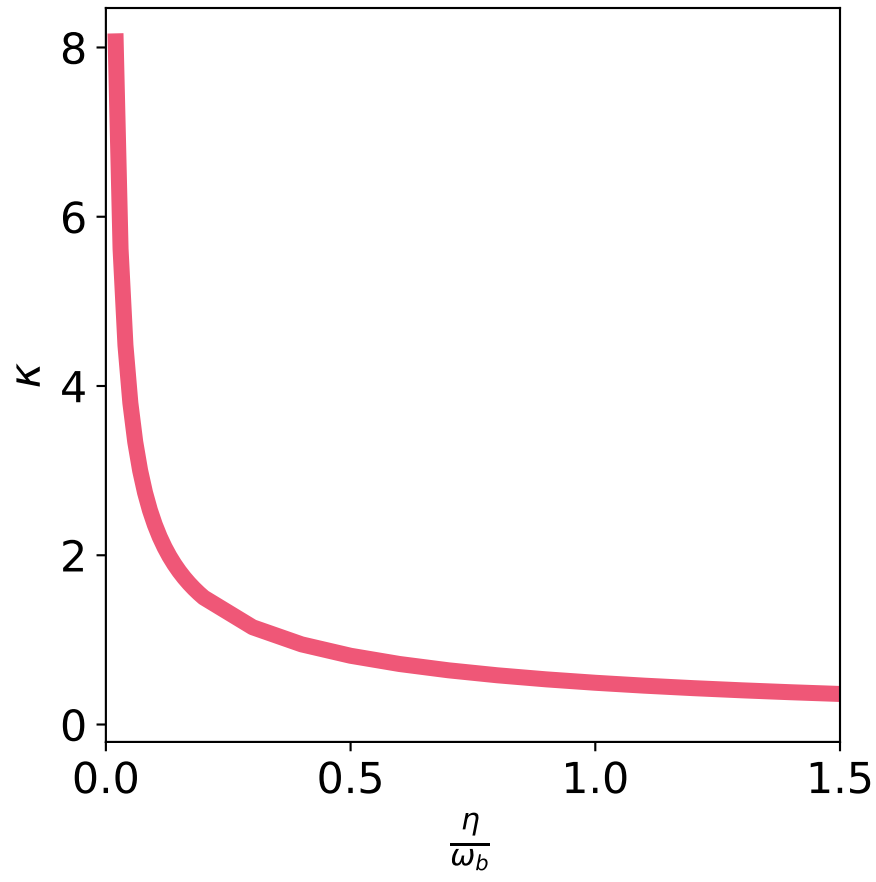

Supplement: Supplementary file 4 — Source Data [file 41467_2023_38368_MOESM4_ESM.zip › Data-VSC-HEOM-main/S3/S3b.pdf]

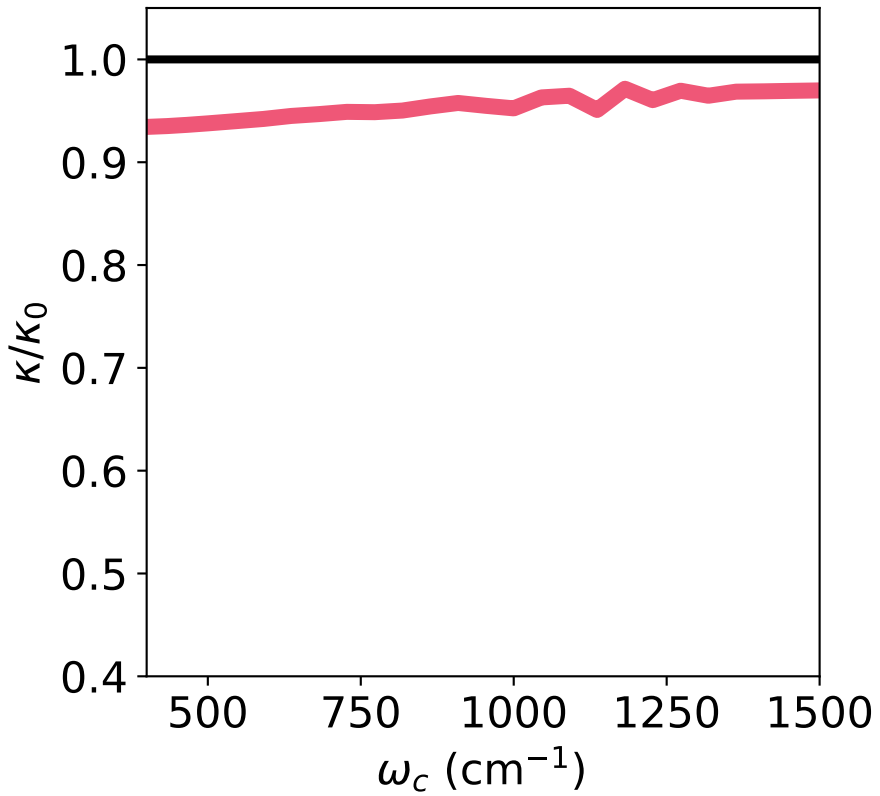

Supplement: Supplementary file 4 — Source Data [file 41467_2023_38368_MOESM4_ESM.zip › Data-VSC-HEOM-main/S3/S3c.pdf]

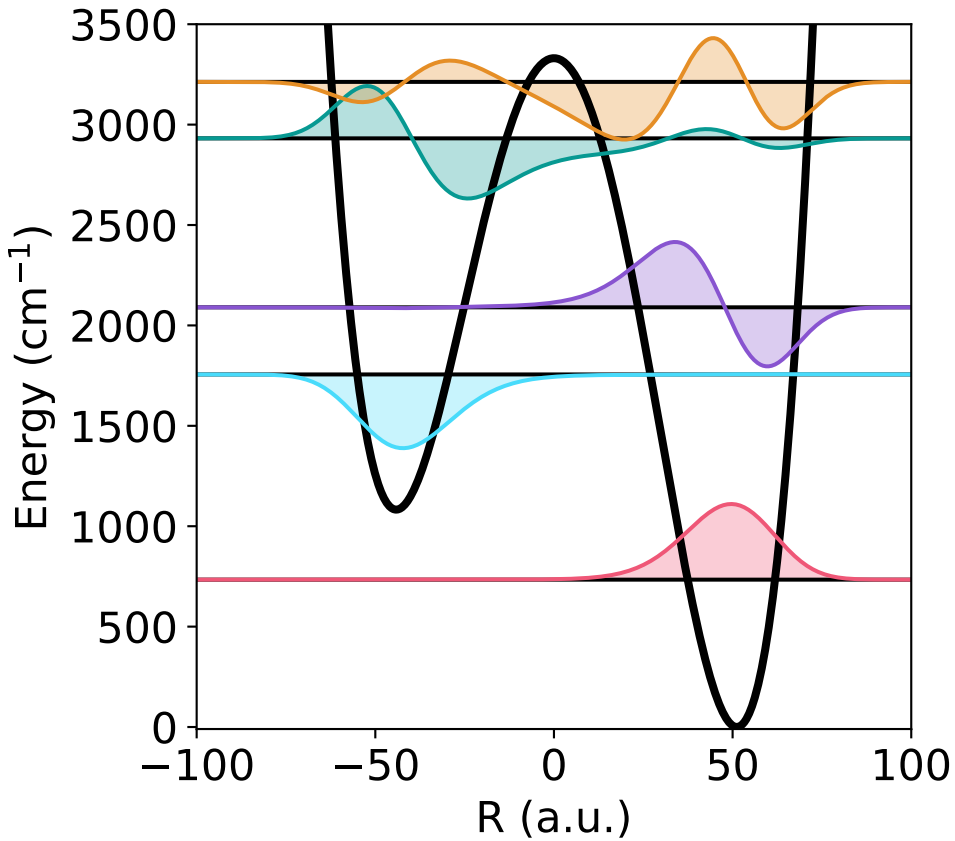

Supplement: Supplementary file 4 — Source Data [file 41467_2023_38368_MOESM4_ESM.zip › Data-VSC-HEOM-main/S4/S4a.pdf]

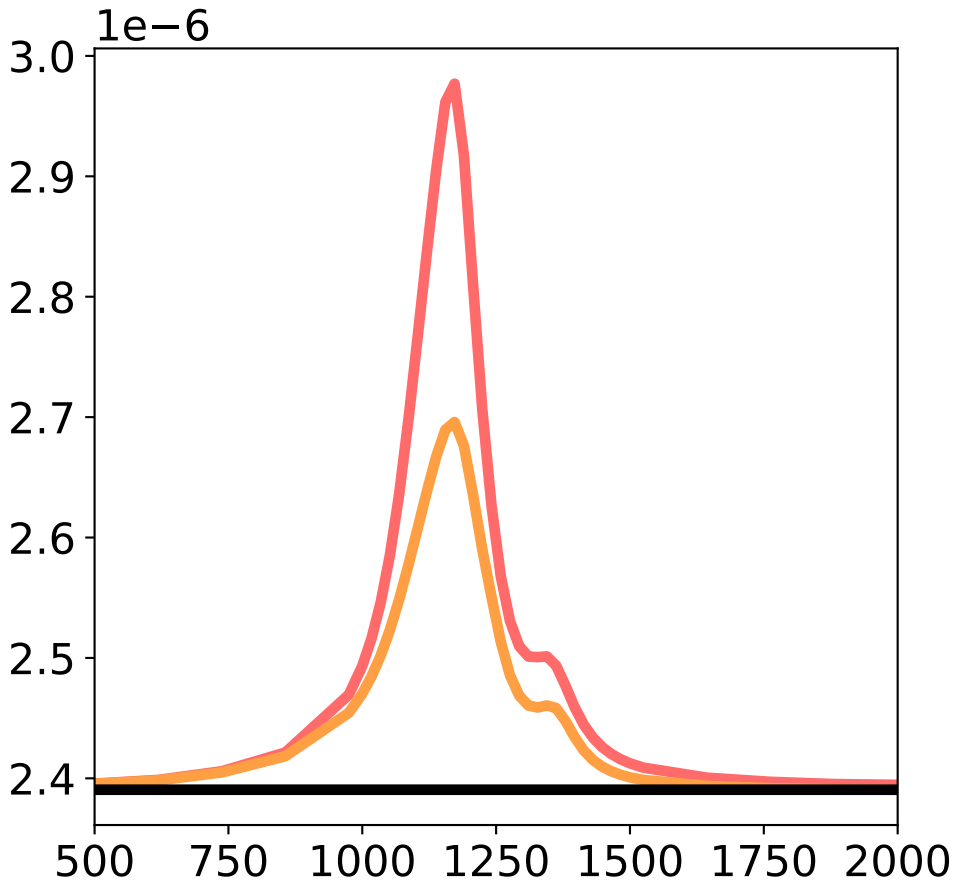

Supplement: Supplementary file 4 — Source Data [file 41467_2023_38368_MOESM4_ESM.zip › Data-VSC-HEOM-main/S4/S4b.pdf]

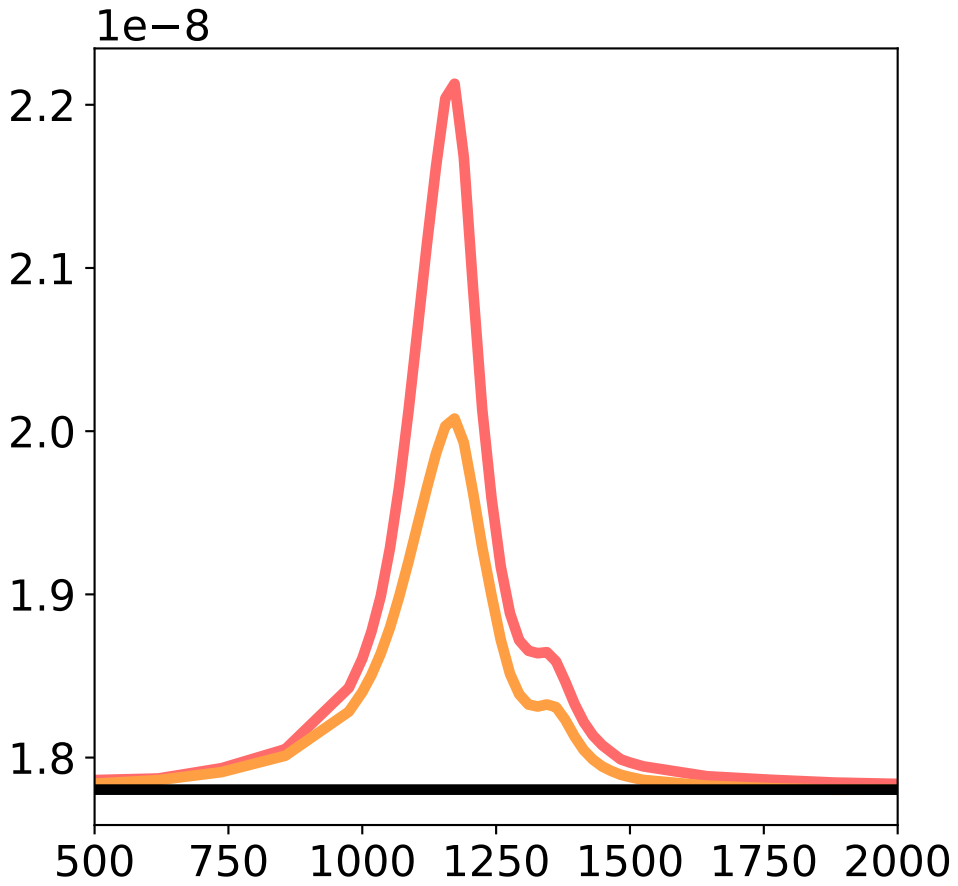

Supplement: Supplementary file 4 — Source Data [file 41467_2023_38368_MOESM4_ESM.zip › Data-VSC-HEOM-main/S4/S4c.pdf]

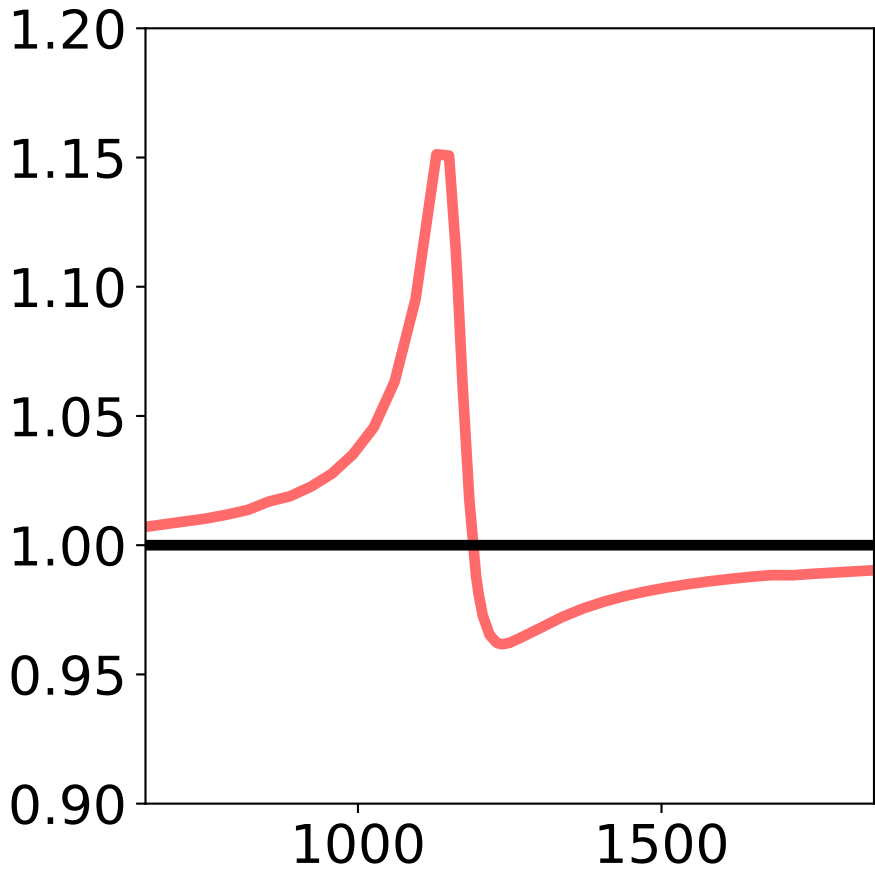

Supplement: Supplementary file 4 — Source Data [file 41467_2023_38368_MOESM4_ESM.zip › Data-VSC-HEOM-main/S5/S5b.pdf]

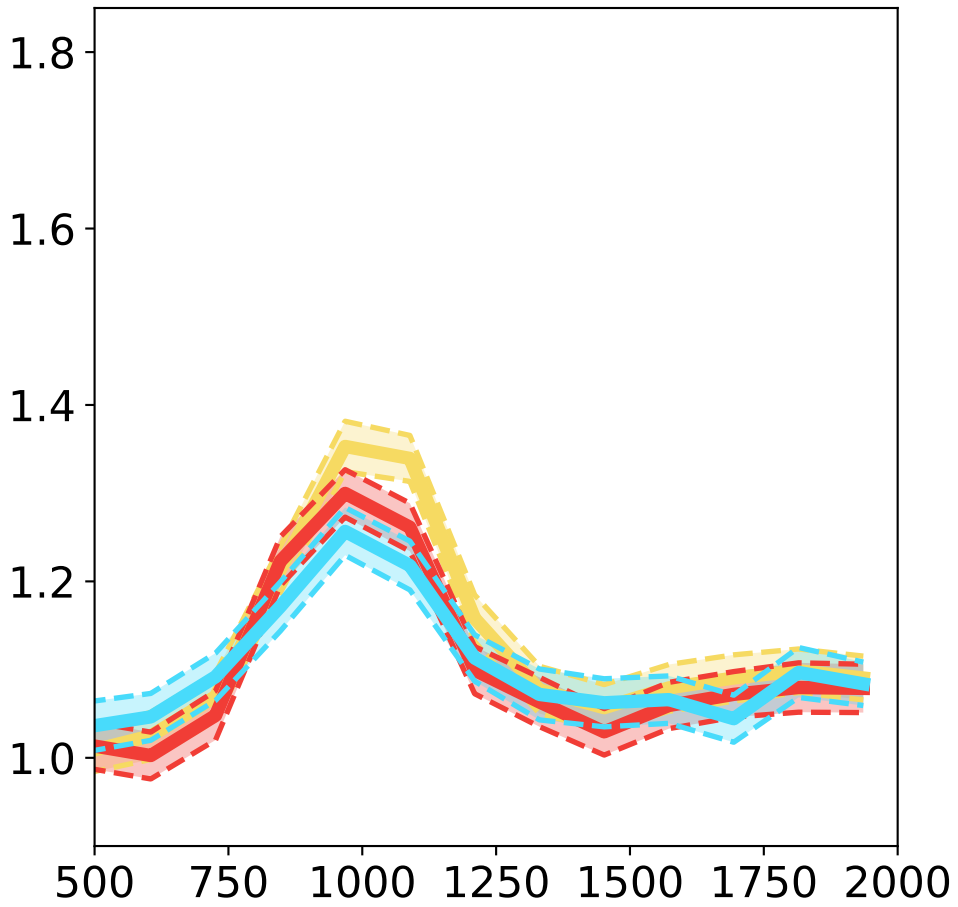

Supplement: Supplementary file 4 — Source Data [file 41467_2023_38368_MOESM4_ESM.zip › Data-VSC-HEOM-main/S6/S6.pdf]

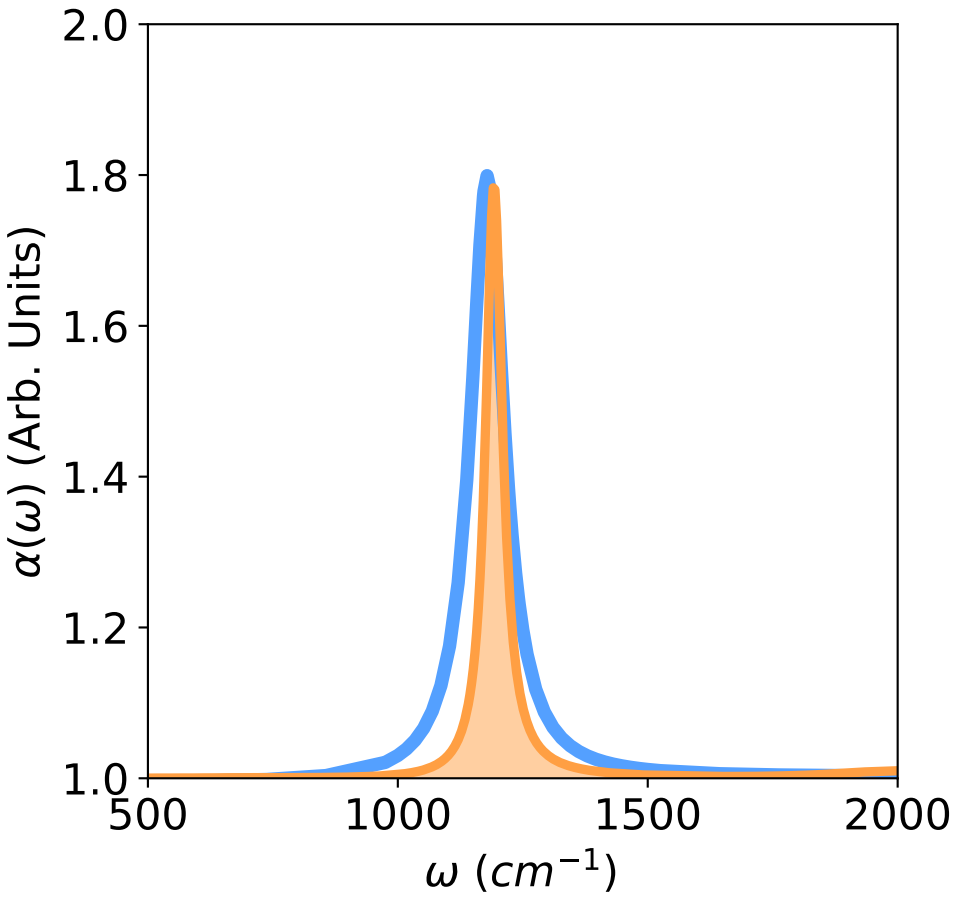

Supplement: Supplementary file 4 — Source Data [file 41467_2023_38368_MOESM4_ESM.zip › Data-VSC-HEOM-main/S7/S7a.pdf]

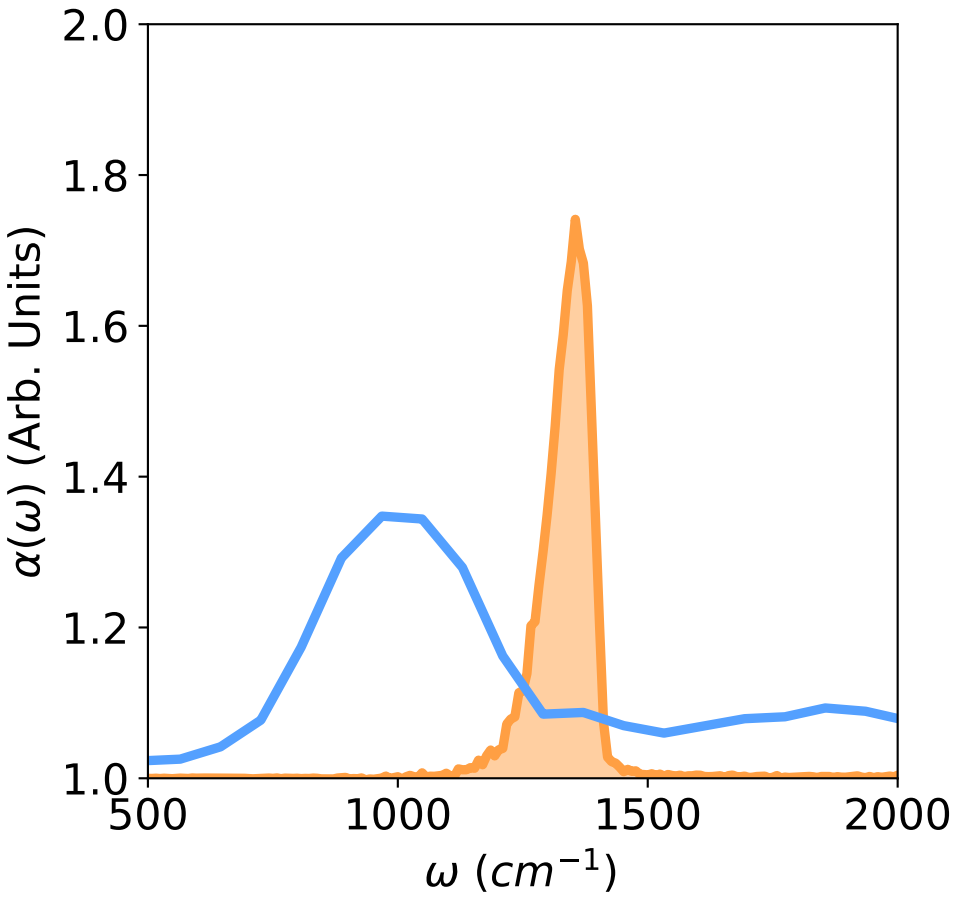

Supplement: Supplementary file 4 — Source Data [file 41467_2023_38368_MOESM4_ESM.zip › Data-VSC-HEOM-main/S7/S7b.pdf]
