# Supplementary material for: Quantum dynamical effects of vibrational strong coupling in chemical reactivity
Source: Nat Commun. 2023 May 12;14:2733. doi: 10.1038/s41467-023-38368-x (PMC10182063; doi:10.1038/s41467-023-38368-x)
Supplement: Supplementary file 4 — Source Data [file 41467_2023_38368_MOESM4_ESM.zip › Data-VSC-HEOM-main/S8/S8b.pdf]

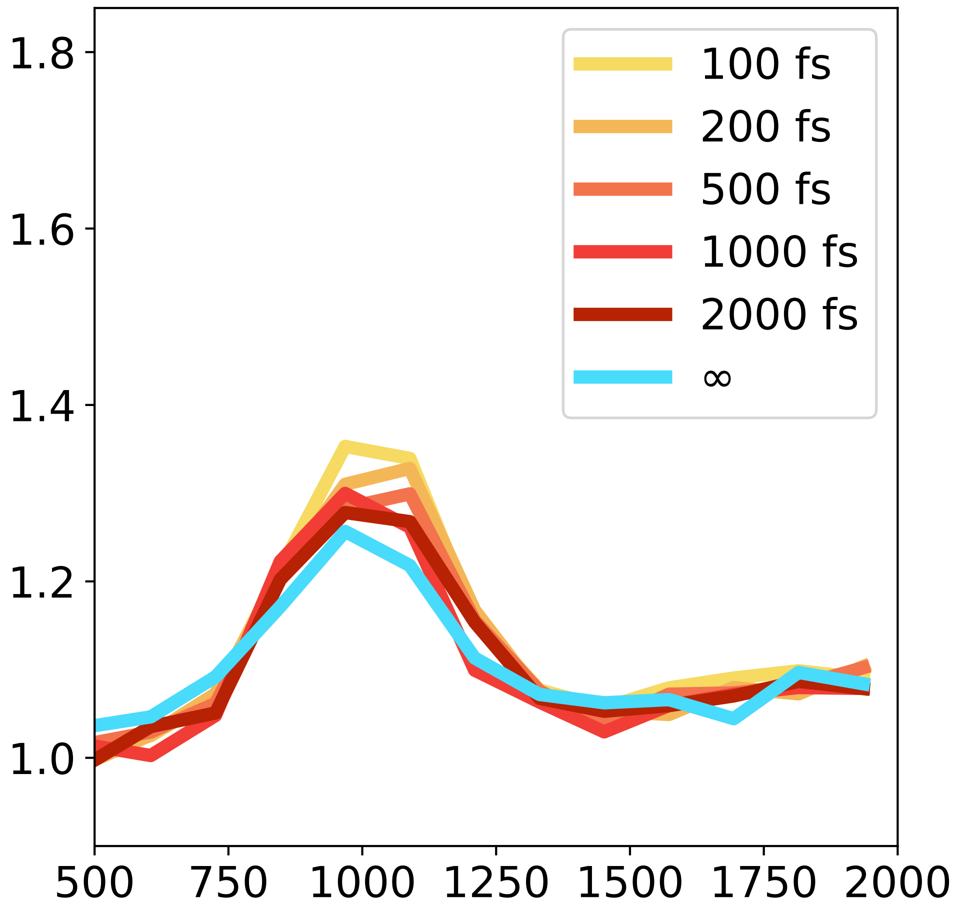

1.8
1.6
1.4
1.2
1.0
500
750
1000
1250
1500
1750
2000
100 fs
200 fs
500 fs
1000 fs
2000 fs
∞
