# Supplementary material for: Quantum dynamical effects of vibrational strong coupling in chemical reactivity
Source: Nat Commun. 2023 May 12;14:2733. doi: 10.1038/s41467-023-38368-x (PMC10182063; doi:10.1038/s41467-023-38368-x)
Supplement: Supplementary file 4 — Source Data [file 41467_2023_38368_MOESM4_ESM.zip › Data-VSC-HEOM-main/S8/S8f.pdf]

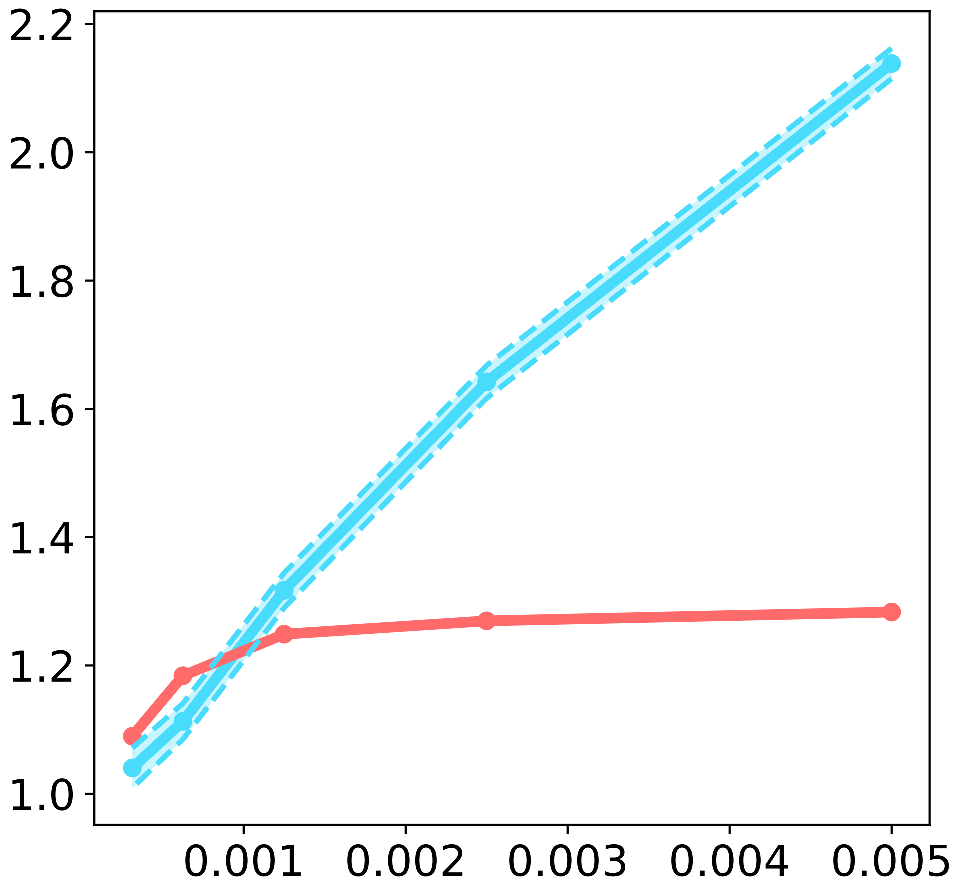
2.2
2.0
1.8
1.6
1.4
1.2
1.0
0.001
0.002
0.003
0.004
0.005
