# Supplementary material for: Quantum dynamical effects of vibrational strong coupling in chemical reactivity
Source: Nat Commun. 2023 May 12;14:2733. doi: 10.1038/s41467-023-38368-x (PMC10182063; doi:10.1038/s41467-023-38368-x)
Supplement: Supplementary file 4 — Source Data [file 41467_2023_38368_MOESM4_ESM.zip › Data-VSC-HEOM-main/S9/S9a.pdf]

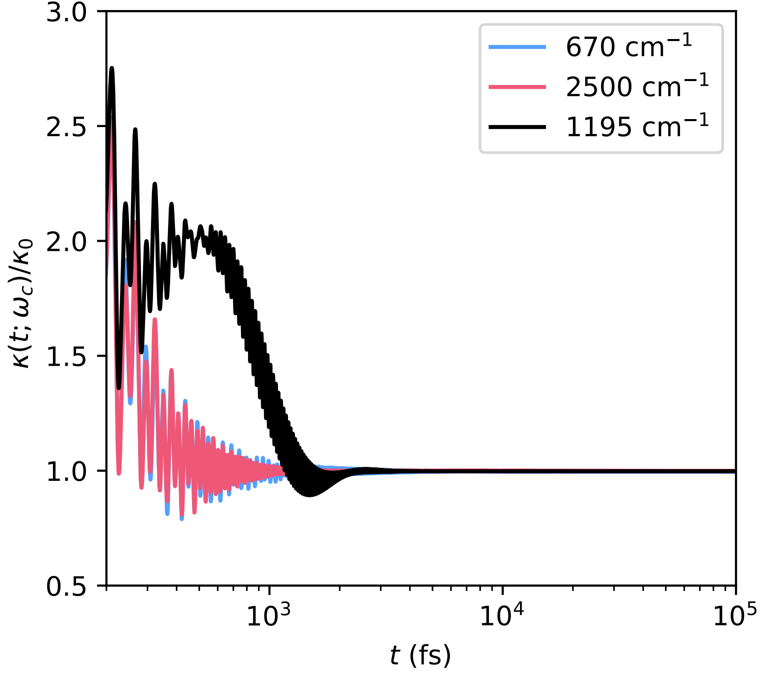
3.0
2.5
2.0
1.5
1.0
0.5
10^3
10^4
10^5
t (fs)
$\kappa(t;\omega_c)/\kappa_0$
670 cm^{-1}
2500 cm^{-1}
1195 cm^{-1}
