# Supplementary material for: Quantum dynamical effects of vibrational strong coupling in chemical reactivity
Source: Nat Commun. 2023 May 12;14:2733. doi: 10.1038/s41467-023-38368-x (PMC10182063; doi:10.1038/s41467-023-38368-x)
Supplement: Supplementary file 4 — Source Data [file 41467_2023_38368_MOESM4_ESM.zip › Data-VSC-HEOM-main/S9/S9b.pdf]

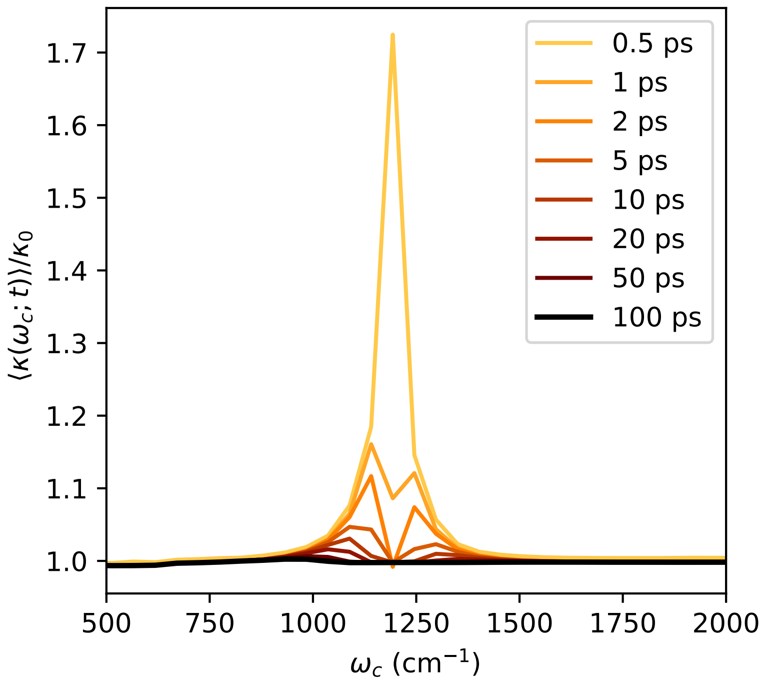

0.5 ps
1 ps
2 ps
5 ps
10 ps
20 ps
50 ps
100 ps
1.7
1.6
1.5
1.4
1.3
1.2
1.1
1.0
$\langle \kappa(\omega_c; t) \rangle / \kappa_0$
500
750
1000
1250
1500
1750
2000
ω_c (cm^{-1})
